# Conserved Functions of *Orthohepadnavirus* X Proteins to Inhibit Type-I Interferon Signaling

**DOI:** 10.3390/ijms25073753

**Published:** 2024-03-28

**Authors:** Amonrat Choonnasard, Maya Shofa, Tamaki Okabayashi, Akatsuki Saito

**Affiliations:** 1 Department of Veterinary Science, Faculty of Agriculture, University of Miyazaki, Miyazaki 889-2192, Japan; amonrat_choonnasard@med.miyazaki-u.ac.jp (A.C.); shofamaya@gmail.com (M.S.); okbys81@cc.miyazaki-u.ac.jp (T.O.); 2 Graduate School of Medicine and Veterinary Medicine, University of Miyazaki, Miyazaki 889-1692, Japan; 3 Center for Animal Disease Control, University of Miyazaki, Miyazaki 889-2192, Japan

**Keywords:** *Orthohepadnavirus*, X protein, domestic cat hepadnavirus (DCH), hepatitis B virus (HBV), interferon-β signaling pathway

## Abstract

*Orthohepadnavirus* causes chronic hepatitis in a broad range of mammals, including primates, cats, woodchucks, and bats. Hepatitis B virus (HBV) X protein inhibits type-I interferon (IFN) signaling, thereby promoting HBV escape from the human innate immune system and establishing persistent infection. However, whether X proteins of *Orthohepadnavirus* viruses in other species display a similar inhibitory activity remains unknown. Here, we investigated the anti-IFN activity of 17 *Orthohepadnavirus* X proteins derived from various hosts. We observed conserved activity of *Orthohepadnavirus* X proteins in inhibiting TIR-domain-containing adaptor protein inducing IFN-β (TRIF)-mediated IFN-β signaling pathway through TRIF degradation. X proteins from domestic cat hepadnavirus (DCH), a novel member of *Orthohepadnavirus*, inhibited mitochondrial antiviral signaling protein (MAVS)-mediated IFNβ signaling pathway comparable with HBV X. These results indicate that inhibition of IFN signaling is conserved in *Orthohepadnavirus* X proteins.

## 1. Introduction

Hepatitis B virus (HBV) (family: *Hepadnaviridae*, genus: *Orthohepadnavirus*) affects >248 million individuals worldwide, and is the major cause of chronic liver disease and liver cancer in humans [1]. Viruses in the *Orthohepadnavirus* genus infect mammals, including primates, cats, woodchucks, and bats [2]. *Orthohepadnavira* exhibit a partially double-stranded DNA genome, ranging between 3.0 and 3.4 kb, encoding four viral (i.e., core, polymerase, surface, and X) proteins [2]. HBV is a “stealth virus” because it triggers a minimal immune response, particularly the type-I interferon (IFN) response, during the initial stages of infection [3]. The failure to induce an innate immune response in infected hepatocytes can lead to incomplete clearance of infected hepatocytes and cause chronic infection [4].

The innate immune system acts as the first line of defense against viral infections by triggering pattern recognition receptors (PRRs), including retinoic acid-inducible gene-I (RIG-I)-like receptors (RLRs) and Toll-like receptors (TLRs) [5]. Several proteins are involved in the PRRs pathway, including mitochondrial antiviral signaling (MAVS) [6], cyclic GMP–AMP synthase (cGAS) [7], and stimulator of interferon genes (STING) [6]. In the TLR3/4-dependent pathway, TIR-domain-containing adaptor inducing interferon-beta (TRIF) is responsible for activating TANK-binding kinase 1 (TBK1) and, subsequently, interferon regulatory factor 3 (IRF-3), contributing to IFN production [7]. Increasing evidence indicates that HBV suppresses IFN signaling [8,9]. Several HBV viral proteins, including polymerase (pol) [10], e antigen [11], core protein (HBcAG) [12], splice protein (HBSP) [13], and X protein (HBx) [14], can suppress cellular innate immunity [15]. HBx is a small protein (154 amino acids) that plays a crucial role in HBV replication [16] and infection [17]. In hepatocytes, HBx disrupted the structural maintenance of chromosome 5/6 complex (Smc5/6) in hepatocytes, which is a restriction factor that inhibits HBV transcription, leading to increased viral transcription and development of cellular transformation through impaired homologous recombination repair of DNA double-strand breaks (DSBs) [18]. HBx comprises two functional domains: N- and C-terminal domains [19,20]. The N-terminal is a negative regulatory domain (amino acid residues 1–50) that consists of a strongly conserved initial segment that exhibits transrepressor activity, specifically within residues 21–50 [21]. The functionality of HBx relies on the C-terminal region, containing the transactivation domain (amino acid residues 52–142) [22]. The stability of HBx is associated with 20 amino acids at the C-terminus (134–154) [23]. HBx inhibits IFN-β production by suppressing RIG-I, melanoma differentiation-associated protein (MDA5), and MAVS [24]. Residues Asn118 and Glu119 in HBx play a critical role in HBx-mediated inhibition of RIG-I–MAVS signaling [25]. Furthermore, HBx decreased TRIF expression through the proteasomal degradation of TRIF in hepatoma cells [26].

In 2018, domestic cat hepadnavirus (DCH), a novel member of the genus *Orthohepadnavirus*, was identified from a domestic cat in Australia [27]. Since then, the prevalence of DCH in cats has been investigated in Italy [28], Thailand [29], Malaysia [30], the United Kingdom [31], Japan [32], the USA [33], Hong Kong [34], Taiwan [35], and Türkiye [36]. The prevalence of DCH infection ranged from 0.2% in the USA to 18.5% in Thailand [33,37]. DCH infection is associated with chronic hepatitis and hepatocellular carcinoma in cats, with increased levels of alanine transaminase (ALT) and aspartate transaminase (AST) [35,38], suggesting similarity with HBV infection in humans. We reported that DCH shares the sodium/bile acid cotransporter (NTCP) with HBV as a cellular entry receptor [39]. However, the functional conservation of DCH X proteins is unclear. We aimed to investigate the functional conservation of *Orthohepadnavirus* X protein from other animal species in inhibiting the IFN-β signaling pathway to better understand viral immune evasion strategies.

In this study, we investigated the function of 17 *Orthohepadnavirus* X proteins on the IFN-β signaling pathway. We found that DCH X and other *Orthohepadnavirus* X proteins tested inhibited TRIF-mediated IFN-β signaling. DCH X protein required 20 amino acids at the C-terminus for efficient expression and function, suggesting that this domain is required for protein stability and is conserved between HBV and DCH X proteins. Thus, the inhibitory function of TRIF-mediated IFN-β signaling pathway is conserved in *Orthohepadnavirus* X protein.

## 2. Results

### 2.1. Genetic Characteristics of Orthohepadnavirus X Proteins

**Figure 1:**
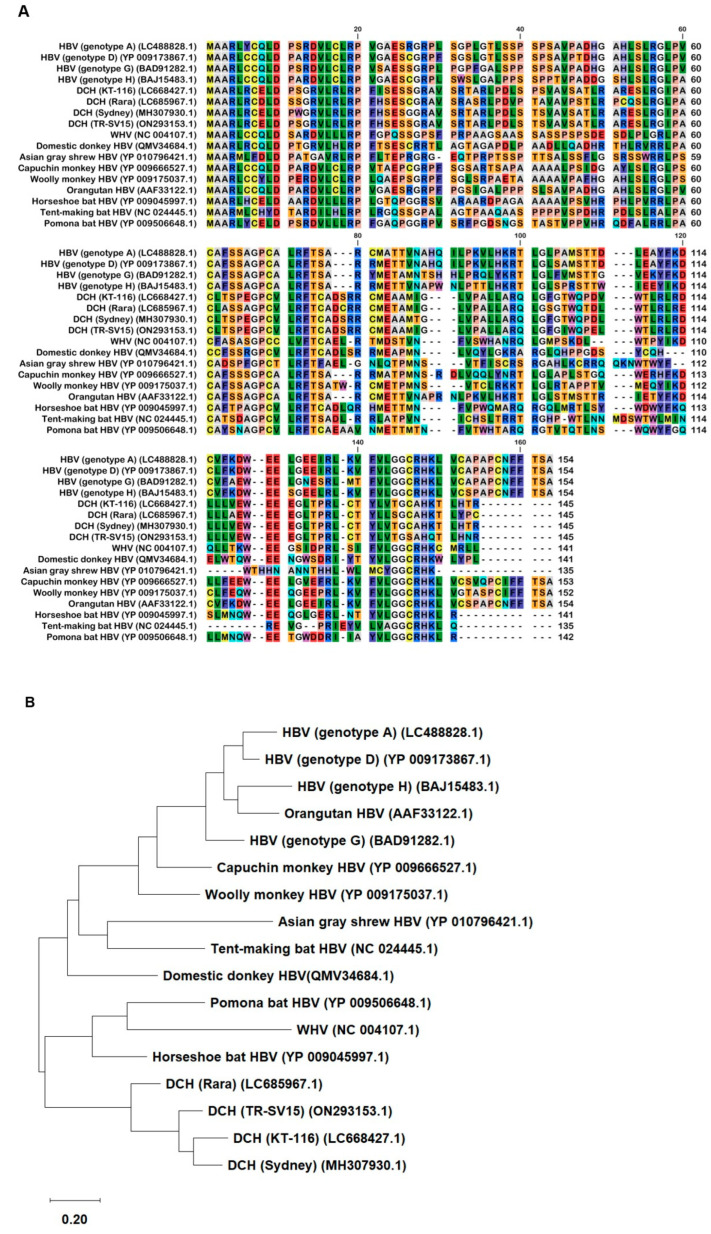
The alignment and phylogenetic tree of *Orthohepadnavirus* X proteins. (**A**) Amino acid alignment of *Orthohepadnavirus* X proteins obtained from public databases. (**B**) The phylogenetic tree was constructed using the MEGA software, and the evolutionary analysis was conducted using the maximum likelihood and neighbor-joining methods based on the Jones–Taylor–Thornton matrix-based model with 1000 bootstrap replicates.

We aligned 17 *Orthohepadnavirus* X proteins, including four strains of human HBV (genotype A, D, G, and H), four strains of DCH (KT-116, Rara, Sydney, and TR-SV15), three strains of bat HBV (pomona bat, horseshoe bat, and tent-making bat), one strain of woodchuck hepatitis virus, and one strain of HBV from the domestic donkey, Asian gray shrew, capuchin monkey, orangutan, and woolly monkey (Figure 1A).

Although X proteins derived from the four DCH strains consist of 145 amino acids (aa), HBV X protein comprises 154 aa. The X protein from DCH (Rara) was distant from the X proteins of other DCH strains (Figure 1B). X proteins from DCH (Rara) and DCH (Sydney: a reference strain of DCH) differed by 14 residues [35]. We observed that the X protein of tent-making bat HBV was more distant from the X proteins of pomona bat HBV and horseshoe bat HBV. X proteins from bat HBV were not closely related, but were genetically similar to HBV strains from other animal species. This suggests viral transmission between bats and other animals (Figure 1B). In addition, the X protein of orangutan HBV is genetically closer to the X protein of HBV than the X proteins of capuchin monkey HBV or woolly monkey HBV. This result suggests that the X protein of ape HBV is more similar to the X proteins of human HBVs than the X proteins of New World monkey HBVs.

To support phylogenetic tree information, we observed the sequence identity of X proteins (Figure S1A). Four strains of HBV X proteins showed a percentage identity (PID) of 100%, the same as four strains of DCH X proteins, while the PID between both groups was 39.87%. Consistent with the result of the phylogenetic tree, we found that the X protein of orangutan HBV has an 87.73% identity compared with the X proteins of HBV, which is higher than the X proteins of capuchin monkey HBV and woolly monkey HBV. Moreover, we investigated the sequence similarity of *Orthohepadnavirus* X proteins (Figure S1B) to identify homologous sequences. DCH X proteins showed a percent similarity with HBV X protein (42.48%) more than other *Orthohepadnavirus* X proteins. This result suggests that HBV and DCH X proteins are closely related.

### 2.2. Both HBV (A) and DCH (KT-116) X Proteins Inhibited IFN-β Signaling Mediated by TRIF, MAVS, and IRF3

**Figure 2:**
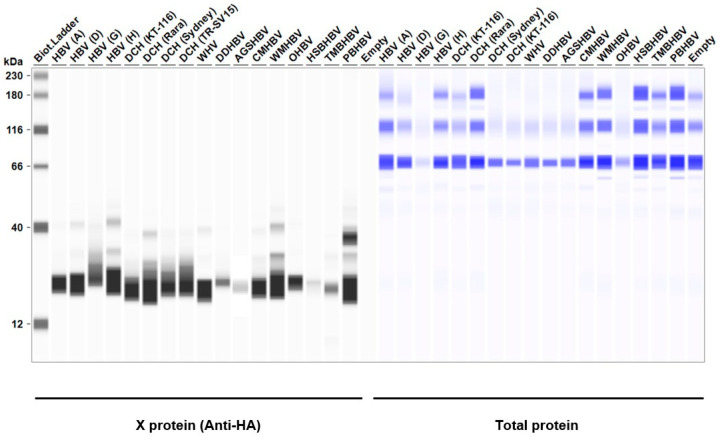
*Orthohepadnavirus* X protein expression levels in Lenti-X 293T cells. The expected HA-tagged X protein sizes ranged between 14.95–16.89 kDa, according to the Protein Molecular Weight website (https://www.bioinformatics.org/sms/prot_mw.html, accessed on 13 March 2024).

Considering the genetic diversity of *Orthohepadnavirus* X proteins across species and strains, we sought to investigate whether the inhibitory effect of HBx on IFN-β induction is conserved in other *Orthohepadnavirus* X proteins. First, we measured the expression level of HA-tagged *Orthohepadnavirus* X proteins in Lenti-X 293T cells with Western blotting. We found comparable expression levels of X proteins in transfected Lenti-X 293T cells (Figure 2). Before testing the inhibitory effect of *Orthohepadnavirus* X proteins, we confirmed that co-transfection with the following plasmids: (1) IFN-β Luc and TRIF, (2) IgK-IFN Luc and MAVS, (3) IgK-IFN Luc and IKKe, (4) interferon-stimulated response element (ISRE) Luc and IRF3, and (5) ISRE Luc and TRIF, significantly induced Firefly luciferase (Figure S2), consistent with other studies [40,41,42].

**Figure 3:**
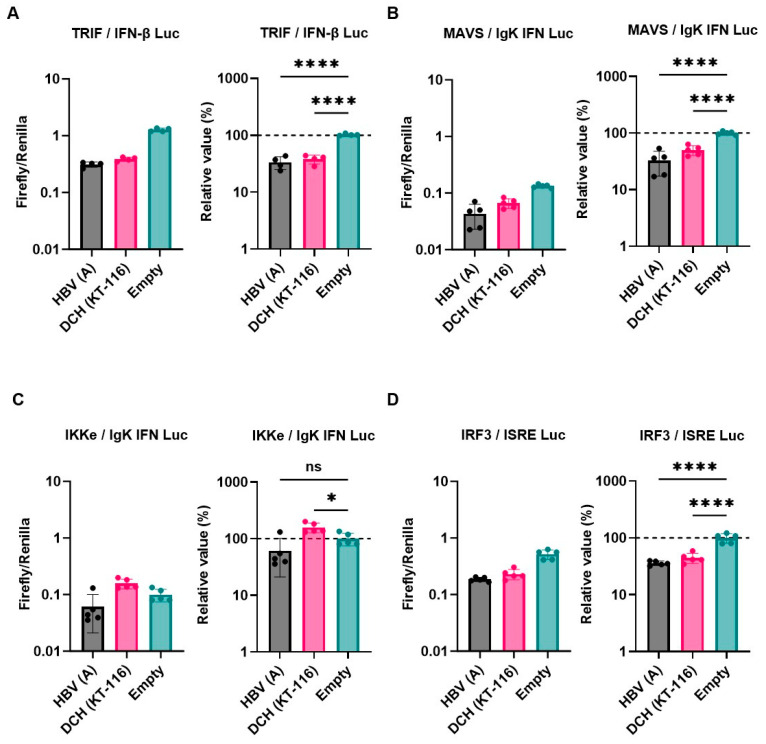
Inhibitory effect of *Orthohepadnavirus* X proteins on IFN-β signaling. (**A**) Inhibitory effect of HBV (genotype A) and DCH (KT-116) upon co-transfection with IFN-β Luc and TRIF plasmids. (**B**) Inhibitory effect of HBV (genotype A) and DCH (KT-116) upon co-transfection with IgK-IFN Luc and MAVS plasmids. (**C**) Effect of HBV (genotype A) and DCH (KT-116) upon co-transfection with IgK-IFN Luc and IKKe plasmids. (**D**) Inhibitory effect of HBV (genotype A) and DCH (KT-116) upon co-transfection with ISRE Luc and IRF3 plasmids. Differences between cells transfected with HBV, DCH X protein plasmids, or an empty plasmid were examined by one-way ANOVA followed by Tukey’s multiple comparison test. **** *p* < 0.0001, * *p* < 0.05, and ns (not significant).

Next, we examined the inhibitory effect of HBV (genotype A) and DCH (KT-116) X proteins on IFN signaling pathways by co-transfection of Lenti-X 293T cells with plasmids expressing X proteins. Consistent with other findings [26], HBx inhibited the induction of Firefly luciferase in cells co-transfected with the following plasmids: IFN-β Luc and TRIF (Figure 3A), IgK-IFN Luc and MAVS (Figure 3B), and ISRE Luc and IRF3 (Figure 3D), but not IgK-IFN Luc and IKKe (Figure 3C). DCH (KT-116) X protein showed similar inhibitory effects with HBV (genotype A) X protein, suggesting functional conservation between HBV (genotype A) and DCH (KT-116) X proteins.

### 2.3. X Proteins Derived from a Range of Orthohepadnavirus sp. Inhibited TRIF-Mediated IFN Signaling

**Figure 4:**
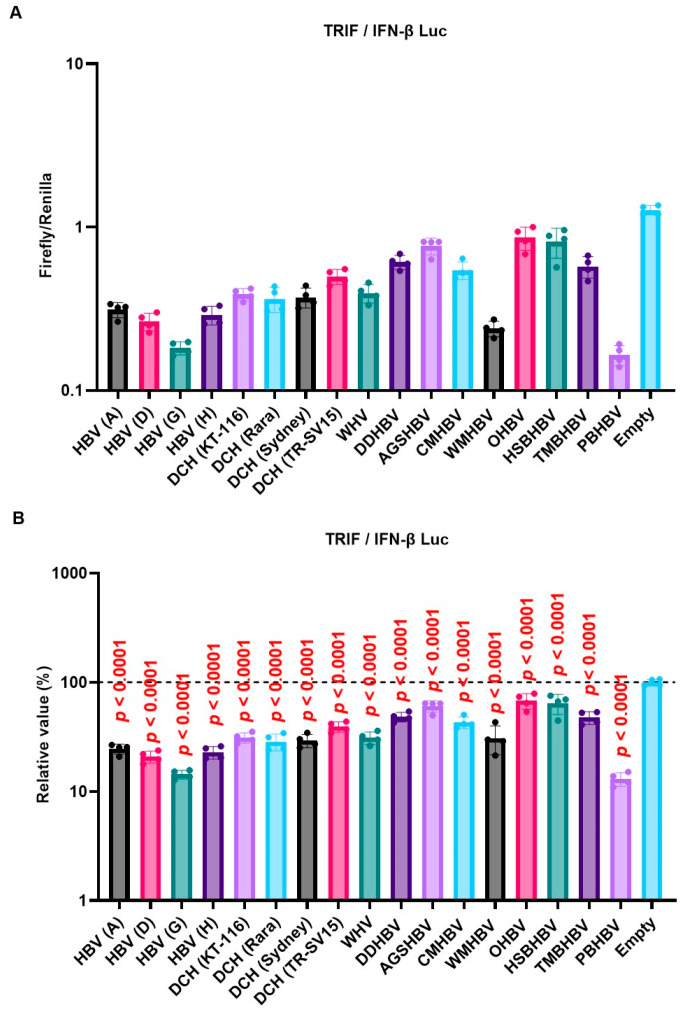
Inhibitory effect of *Orthohepadnavirus* X proteins on TIR-domain-containing adaptor protein inducing IFN-β (TRIF)-mediated IFN-β signaling. (**A**) Raw data of the luciferase reporter assay. The RLU of Firefly luciferase was divided by the RLU of Renilla luciferase. (**B**) Relative value of IFN-β luciferase reporter assay. Differences between cells transfected with plasmids expressing *Orthohepadnavirus* X protein or an empty plasmid were examined by one-way ANOVA followed by Dunnett’s multiple comparison test.

We observed a conserved inhibitory effect of HBV (genotype A) and DCH (KT-116) X proteins on IFN signaling (Figure 3A,B,D). Notably, HBV (genotype A) and DCH (KT-116) X proteins potently inhibited TRIF-mediated IFN-β signaling (Figure 3A). To investigate whether the inhibitory effect of X proteins on TRIF-mediated IFN-β signaling is conserved across *Orthohepadnavirus* sp., Lenti-X 293T cells were co-transfected with plasmids expressing the 17 *Orthohepadnavirus* X proteins with IFN-β Luc and TRIF plasmids (Figure 4). We found that all *Orthohepadnavirus* X proteins significantly inhibited TRIF-mediated IFN-β signaling (Figure 4A,B), suggesting that the inhibitory effect on TRIF-mediated IFN signaling is conserved in X proteins across *Orthohepadnavirus* sp. To further test the inhibitory effect of *Orthohepadnavirus* X proteins, we co-transfected Lenti-X 293T cells with plasmids expressing IFN-β Luc, MAVS, and HBV strains (genotype A, D, G, and H) or DCH strains (KT-116, Rara, Sydney, and TR-SV15) X proteins. Although the inhibitory effect is smaller than on TRIF-mediated IFN-β signaling (Figure S3), *Orthohepadnavirus* X proteins target MAVS-mediated IFN-β signaling.

### 2.4. The C-Terminus Transactivation Domain of X Proteins Plays an Important Role in Stabilizing Protein Expression and Function

**Figure 5:**
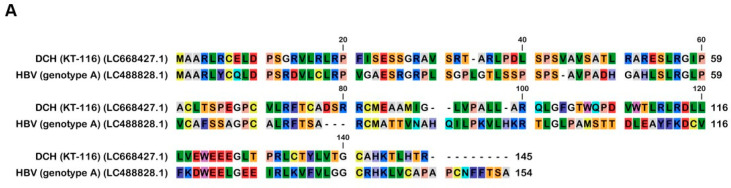

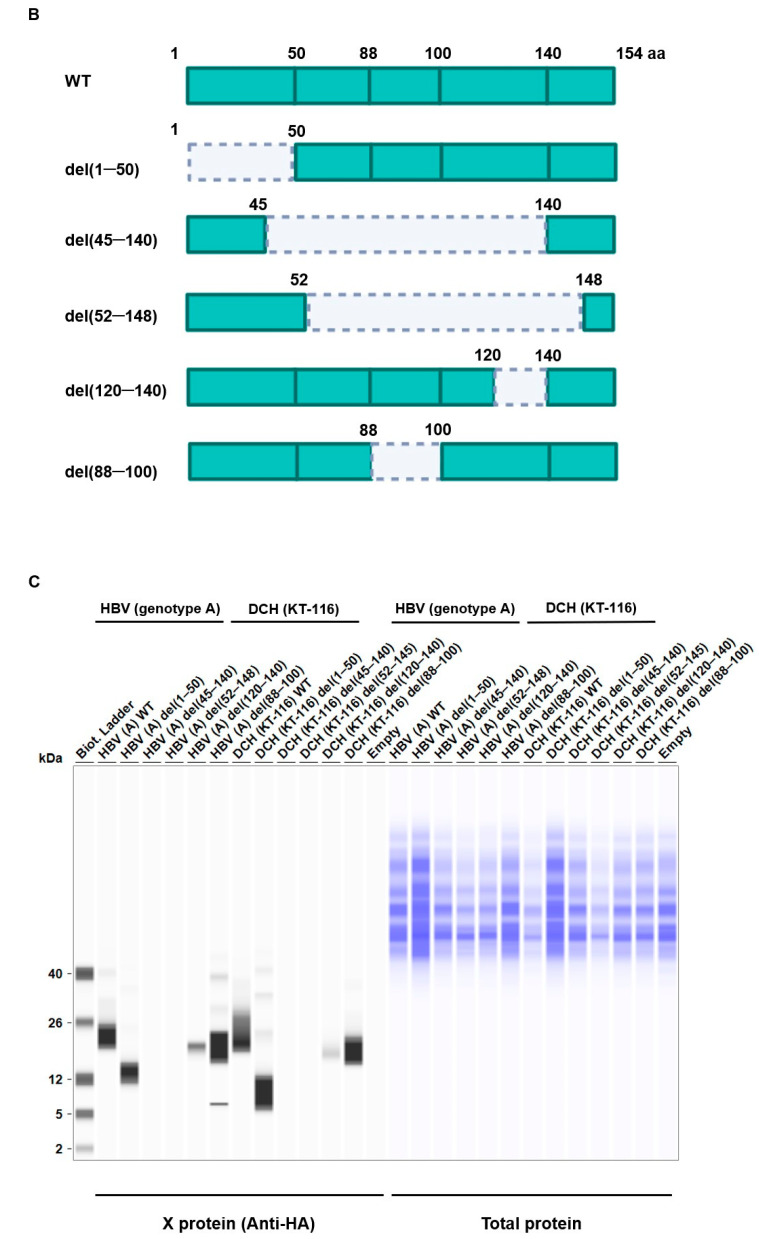

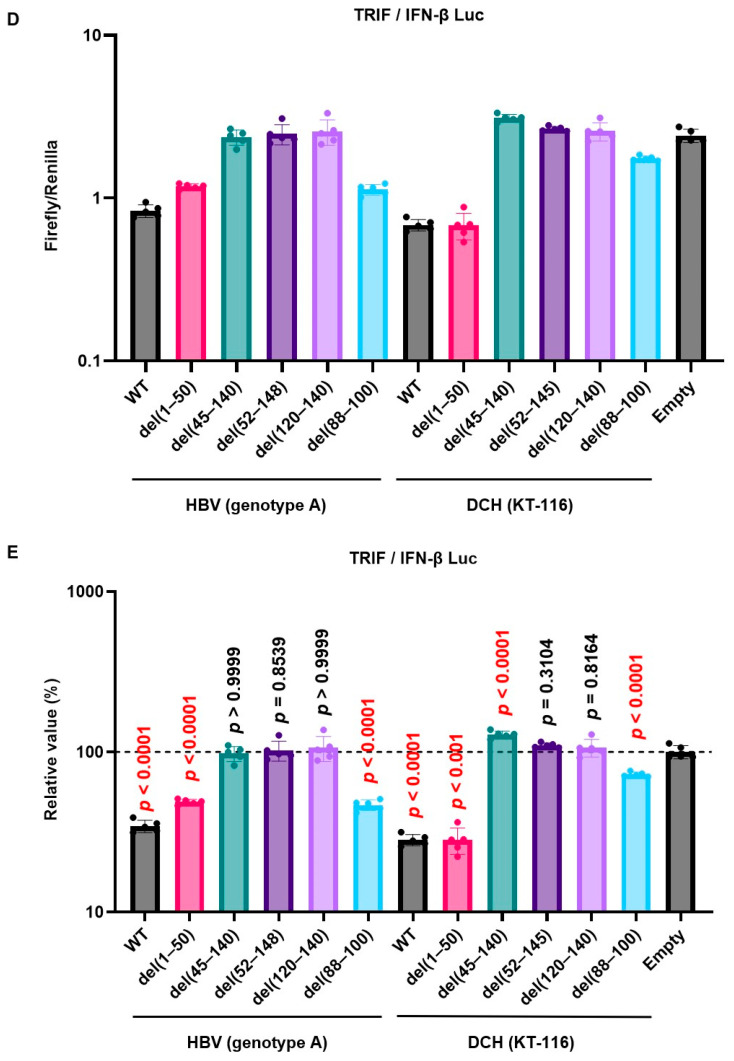
The C-terminus transactivation domain of X protein plays an important role in protein stability and function. (**A**) Amino acid alignment of HBV (genotype A) and DCH (KT-116) X proteins. (**B**) Deletion mutants of X proteins. (**C**) HBV (genotype A) and DCH (KT-116)-derived mutant X protein expression levels in Lenti-X 293T cells. The expected HA-tagged WT and mutant X protein sizes were approximately 16 kDa and ranged between 5.40 and 14.04 kDa, respectively. (**D**) Raw data from the luciferase reporter assay. The RLU of Firefly luciferase was divided by the RLU of Renilla luciferase. (**E**) Relative value of the IFN-β luciferase reporter assay. Differences between cells transfected with *Orthohepadnavirus* X protein plasmids or an empty plasmid were examined by one-way ANOVA followed by Dunnett’s multiple comparison test.

We showed that the inhibitory effect on TRIF-mediated IFN-β signaling is conserved in *Orthohepadnavirus* X proteins (Figure 4A,B). We found genetic variations in *Orthohepadnavirus* X proteins, especially in the transactivation domain (Figure 1). Here, we aimed to identify domains in X proteins that determine inhibitory effects on TRIF-mediated IFN-β signaling. Therefore, we made a series of deletion mutants of HBV (genotype A) and DCH (KT-116) X proteins (Figure 5A,B), as described [43]. We made mutants with deletions in the (1) negative regulatory domain (amino acids (aa) 1–50: del(1–50)), (2) transactivation domain (aa 52–148: del(52–148)), (3) nuclear translocation domain (aa 120–140: del(120–140), (4) region that interacts with the host’s structural maintenance of chromosome 5/6 complex (Smc5/6)(aa 45–140: del(45–140)), and (5) region that interacts with the host’s damaged DNA binding protein 1 (DDB1) (aa 88–100: del(88–100)) (Figure 5B).

The deletion mutant plasmids of HBV (genotype A) and DCH (KT-116) X proteins were used to co-transfect Lenti-X 293T cells with IFN-β Luc and TRIF plasmids. We found significantly lower expression levels of del(52–148) and del(45–140) mutants of HBV (genotype A) X protein in Lenti-X 293T cells (Figure 5C). Although the similarity of the transactivation domains between HBV (genotype A) and DCH (KT-116) X proteins is low, del(52–148) and del(45–140) X protein mutants of DCH (KT-116) X protein shared phenotype with HBV (genotype A) del(52–148) and del(45–140) mutants.

Next, we examined the subcellular localization of X proteins in Lenti-X 293T cells. Wild-type (WT) X proteins of HBV (genotype A) and DCH (KT-116) were primarily localized in the nucleus, but were also detected in the cytoplasm (Figure S4), which is consistent with another study [44]. Mutant X proteins with deletions had similar localization, suggesting that the deletion of N- and C-terminal amino acids of HBV (genotype A) and DCH (KT-116) X proteins minimally affected protein localization in Lenti-X 293T cells.

Consistent with the result of Western blotting, we observed both del(52–148) and del(45–140) X protein mutants of both HBV (genotype A) and DCH (KT-116) failed to inhibit TRIF-mediated IFN-β signaling (Figure 5D,E). These results demonstrated that the transactivation domain at the C-terminus has an important role in stabilizing the expression and function of *Orthohepadnavirus* X proteins.

**Figure 6:**
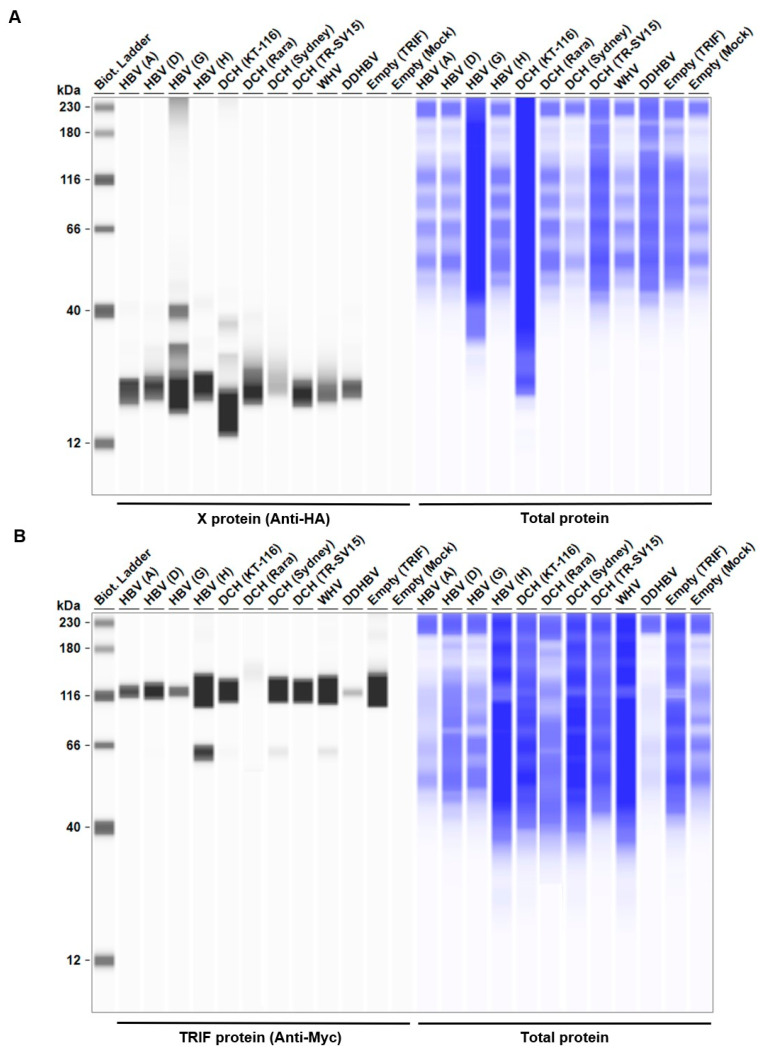

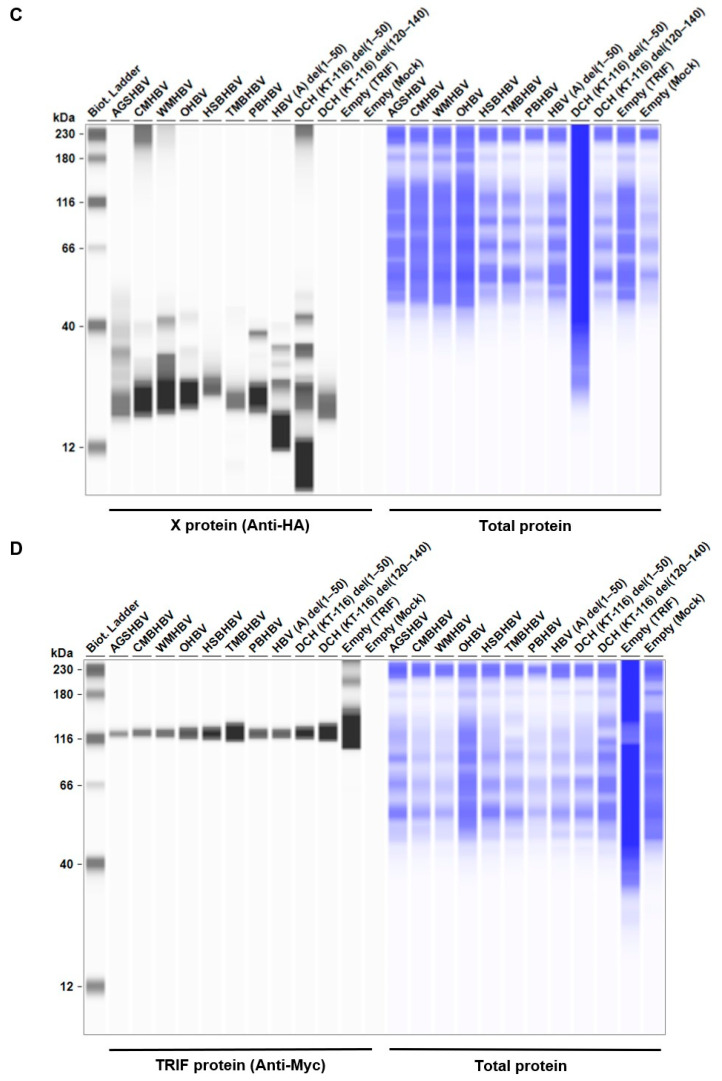

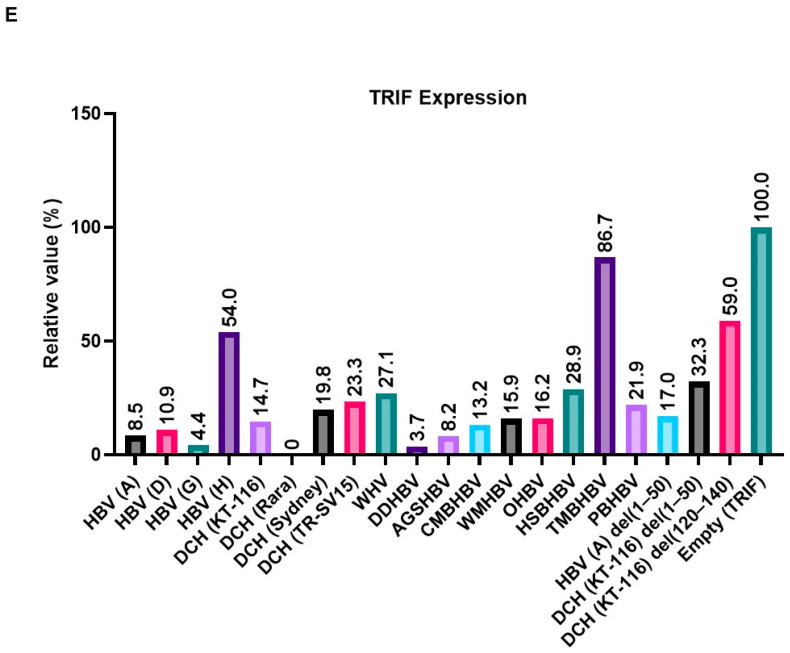
*Orthohepadnavirus* X protein degrades TIR-domain-containing adaptor protein inducing IFN-β (TRIF). (**A**,**C**) HA-tagged *Orthohepadnavirus* X protein expression levels in transfected Lenti-X 293T cells. The expected HA-tagged X protein sizes ranged between 10.64 and 16.89 kDa. (**B**,**D**) Myc-tagged human TRIF expression levels in transfected Lenti-X 293T cells. The expected Myc-tagged human TRIF size was 120 kDa. (**E**) Relative values of TRIF expression. Relative value was calculated from the corrected area of the TRIF band with Compass for Simple Western software version 6.3.0.

To elucidate the mechanism of the inhibitory effect on the TRIF-mediated IFN-β signaling pathway, we co-transfected Lenti-X 293T cells with plasmids expressing TRIF and X proteins from a broad range of species. We found that the expression of X proteins induced 13–100% degradation of TRIF (Figure 6E). Although DCH (Rara) X protein degraded TRIF completely, TMBHBV X protein decreased TRIF expression by ~13% in Lenti-X 293T cells. These results suggest that X protein inhibits TRIF-mediated IFN-β signaling by degrading TRIF.

### 2.5. Variation in the C-Terminus Transactivation Domain of DCH X Protein Determines the Inhibitory Effect on ISRE-Mediated IFN-β Signaling

**Figure 7:**
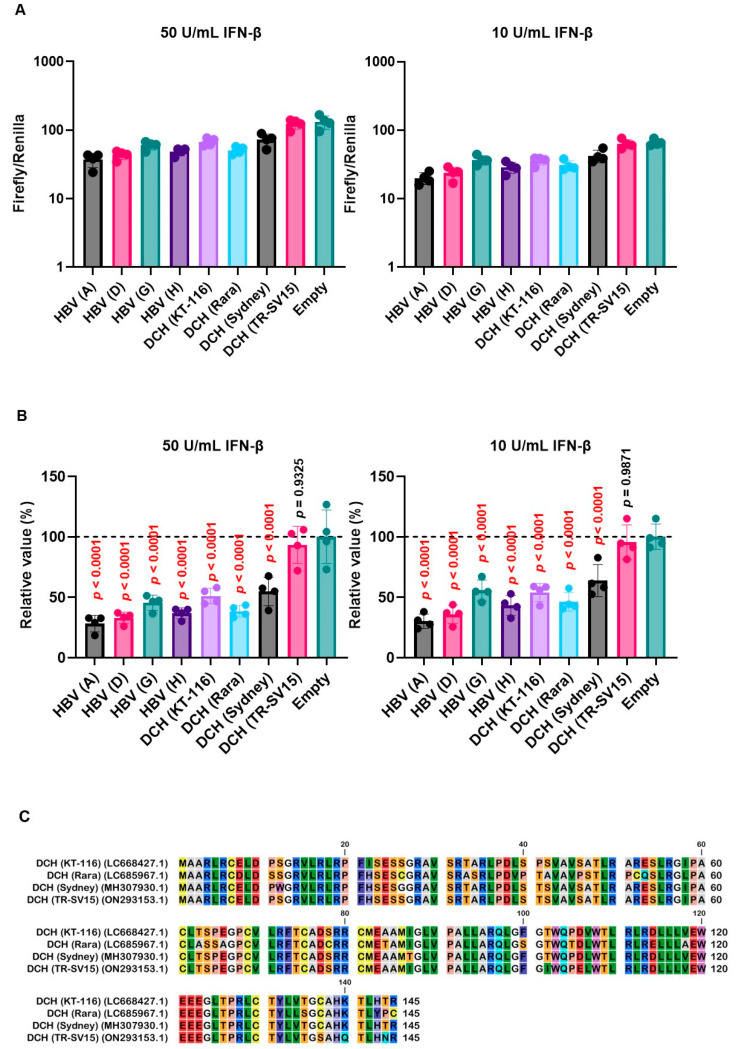

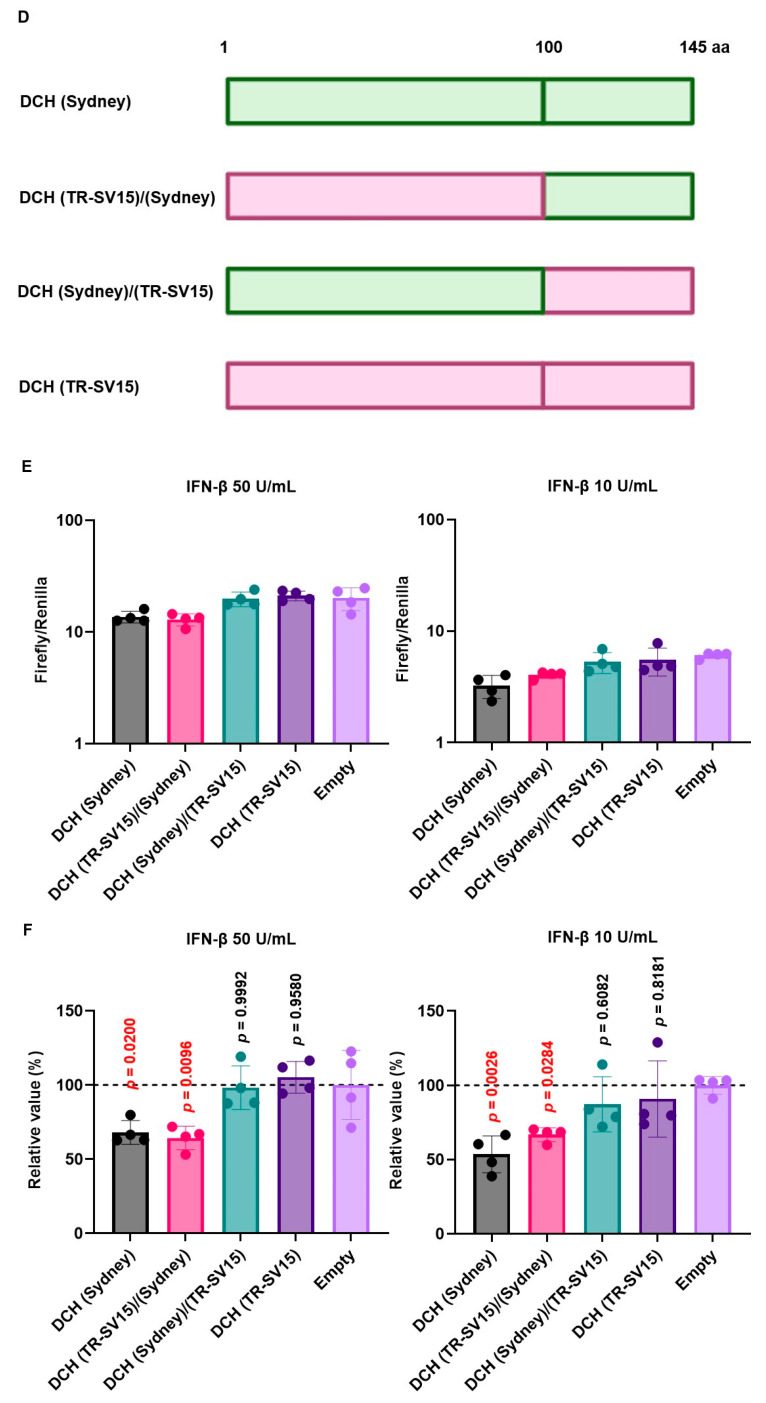
Variations in the C-terminus transactivation domains of DCH X proteins determine inhibitory effects on interferon-stimulated response element (ISRE)-mediated IFN-β signaling. (**A**,**E**) Raw data of the luciferase reporter assay. The RLU of Firefly luciferase was divided by the RLU of Renilla luciferase. (**B**,**F**) Relative value of IFN-β luciferase reporter assay. Differences between cells transfected with *Orthohepadnavirus* X protein plasmids or an empty plasmid were examined by one-way ANOVA followed by Dunnett’s multiple comparison test. (**C**) Amino acid alignment of DCH (KT-116), DCH (Rara), DCH (Sydney), and DCH (TR-SV15) X proteins. (**D**) Schematic representation of chimeric X proteins between DCH (Sydney) and DCH (TR-SV15) X proteins.

To evaluate the effect of *Orthohepadnavirus* X proteins in inhibiting ISRE-mediated IFN-β signaling, we used 293T-ISRE-luc2 cells. We confirmed that 293T-ISRE-luc2 cells could be stimulated with recombinant human IFN-β, leading to a significant induction of luciferase (Figure S5). Next, we transfected 293T-ISRE-luc2 cells with plasmids expressing *Orthohepadnavirus* X proteins to test the inhibitory effect of *Orthohepadnavirus* X proteins on ISRE-mediated IFN-β signaling. Most X proteins, except DCH (TR-SV15) X, suppressed ISRE-mediated IFN-β signaling (Figure 7A,B). Alignment of the X proteins of the four DCH strains showed that in DCH (TR-SV15) X protein, five residues (positions 102, 106, 137, 140, and 144) differed in the C-terminus transactivation domain compared with the other three strains (Figure 7C). This indicates that these five amino acids might be responsible for the impaired inhibition of ISRE-mediated IFN-β signaling.

To address this possibility, we generated chimeric X proteins between DCH (Sydney) and DCH (TR-SV15) (Figure 7D). The chimeric DCH X proteins were efficiently expressed in 293T-ISRE-luc2 cells compared with WT proteins (Figure S6). Although DCH (Sydney)/(TR-SV15) X protein failed to inhibit ISRE-mediated IFN-β signaling, DCH (TR-SV15)/(Sydney) X protein showed potent inhibition of ISRE-mediated IFN-β signaling (Figure 7E,F). These results suggest that variations in the C-terminus transactivation domains of DCH X proteins determine the inhibitory effect on ISRE-mediated IFN-β signaling.

## 3. Discussion

In this study, we demonstrated that *Orthohepadnavirus* X protein inhibits TRIF-mediated IFN-β signaling, mainly through TRIF degradation. This inhibitory activity is conserved across *Orthohepadnavirus* X proteins. The C-terminus transactivation domains have an important role in stabilizing the expression and function of *Orthohepadnavirus* X protein to inhibit TRIF-mediated IFN-β signaling.

In 1991, the domains of HBx were characterized; the domain between residues 103 and 117 of the C-terminus transactivation domain was found to be important for a fully functional HBx [45]. Here, we aligned 17 *Orthohepadnavirus* X proteins and found high genetic diversity at the C-terminus transactivation domains (Figure 1A). The phylogenetic tree showed that DCH X proteins are more distant from HBx than X proteins from other species (Figure 1B). Nevertheless, DCH X proteins shared a function with HBx to inhibit IFN-β signaling (Figure 3A,B,D). We recently reported that DCH shares the cell entry molecule, sodium/bile acid cotransporter, with HBV [39]. However, DCH preS1 is genetically closer to HBV preS1 than woodchuck hepatitis virus or Arctic ground squirrel HBV [39]. Understanding the similarities and differences between HBV and DCH is critical for using DCH as a model for HBV research.

DCH (KT-116) X protein modulated the innate immune response, including the TLR3 and RIG-I-like receptor response, by inhibiting TRIF (Figure 3A), MAVS (Figure 3B), and IRF-3 signaling (Figure 3D), similar to HBx. HBx was reported to inhibit MAVS activation by aggregation without affecting the expression of MAVS and RIG-I [25]. By contrast, TRIF activation was inhibited by inducing TRIF degradation [26]. Consistently, our results indicate that *Orthohepadnavirus* X protein inhibits TRIF-mediated IFN-β signaling by degrading TRIF (Figure 6B,D). Interestingly, X proteins derived from TMBHBV (13%) and HBV (genotype H) (50%) showed weaker TRIF degradation activity than other X proteins. Nevertheless, these X proteins inhibited TRIF-mediated IFN-β signaling (Figure 4B). Although DCH (KT-116) del(120–140) X protein decreased TRIF expression (~40%) (Figure 6E), it failed to inhibit TRIF-mediated IFN-β signaling (Figure 5D,E), suggesting a TRIF degradation-independent mechanism to block signaling.

HBx was reported to induce ubiquitin-mediated protein degradation [46] and TRIF degradation through a proteasomal pathway in hepatoma cells [26]. In this study, *Orthohepadnavirus* X proteins from broad range of species exhibited a conserved mechanism for TRIF degradation to perturb innate immunity. Our results increase our understanding of *Orthohepadnavirus* X protein-mediated suppression of TLR signaling. Consistently, HBV was reported to utilize X protein to avoid the innate immune system, establishing chronic infection in the host [14,24,47,48].

The 3D structure of *Orthohepadnavirus* X protein is unsolved. However, it has two functional domains, including an N-terminal negative regulatory and a C-terminal transactivation domain [20]. The N-terminal region (aa 1–50) contains a highly conserved region (aa 1–20) and a Ser/Pro-rich region (aa 21–50) that is essential for negative regulatory effects and association of the regulatory domain [21]. C-terminal residues 58–119 are associated with signal transduction to the nucleus [49], residues 120–140 play an important role in nuclear transactivation, and the last 20 amino acids (aa 134–154) are involved in protein stability [22,23]. Consistently, we found that the deletion of residues 120–140 impaired the stability of the X protein (Figure 5C) and the degradation activity of TRIF (Figure 6D), leading to failed inhibition of IFN signaling (Figure 5D,E). Although the amino acid length of DCH X proteins is nine amino acids shorter than HBx, and there is high genetic variation at position 120–140 (Figure 5A), our results suggest that the C-terminal domains of DCH X proteins have conserved function with HBx (regarding protein stability and inhibition of IFN signaling).

The localization of HBx was shown to depend on its expression level: low expression leads to nuclear localization [44]; high expression leads to cytoplasmic localization and abnormal mitochondrial distribution [44]. In this study, *Orthohepadnavirus* X proteins from various species showed conserved localization primarily in the nucleus, but also localized to the cytoplasm (Figure S4A). Deletion at the N- or C-terminal domain marginally affected the localization of HBV and DCH X proteins (Figure S4B). These observations suggest that the C-terminal domain is important for the inhibitory effect of the X proteins, but does not determine their cellular localization.

X proteins derived from HBV and DCH showed an inhibitory activity on ISRE-mediated IFN-β signaling, except DCH (TR-SV15) (Figure 7A,B). Alignment of X proteins derived from the four DCH strains showed that DCH (TR-SV15) had five different amino acids in the C-terminus transactivation domain (Figure 7C). Analysis using chimeric X proteins between DCH (Sydney) and DCH (TR-SV15) proteins suggested that genetic variations in the C-terminus of DCH X protein determine its inhibitory effect on ISRE-mediated IFN-β signaling (Figure 7E,F). Further analyses are required to identify specific residue(s) that determine inhibitory activity.

Several host and viral factors, such as host age and gender, viral load, and viral genotype, can influence the IFN response during infection and IFN treatment [50]. Among viral factors, the X protein is one of the important factors in the IFN response, because it not only inhibits TLR3–TRIF and RIG-I signaling, but also suppresses IFN induction by suppressing the transcription of tripartite motif 22 in a mouse model, primary human hepatocytes, and human liver tissues [51]. The mechanism for the loss of inhibitory effect of DCH (TR-SV15) on ISRE-mediated IFN-β signaling is unclear. Therefore, we must elucidate the determinant(s) that can affect the IFN response in DCH-infected cats. Our results provide clues to improve the efficiency of IFN therapy in cats with chronic hepatitis.

A limitation of this study is that we used a luciferase reporter system to probe the interaction between *Orthohepadnavirus* X protein and human-derived molecules involved in IFN signaling. We must investigate whether (1) *Orthohepadnavirus* X proteins a show similar effect on molecules derived from other species, and (2) our results can be reproduced in primary cells. Lastly, we must investigate the immunopathogenesis induced by *Orthohepadnavirus* X proteins, especially DCH. These findings will contribute to developing therapeutic guidelines to counteract DCH infection effectively.

In conclusion, our results revealed that DCH X proteins and other *Orthohepadnavirus* X proteins inhibit TRIF-mediated IFN-β signaling by degrading TRIF, suggesting that this mechanism is a conserved function of *Orthohepadnavirus* X proteins to perturb the host’s innate immune response. Our findings show that the C-terminus domains of HBV and DCH X proteins are important for protein stability and inhibitory function. The results of this study deepen our understanding of the function of *Orthohepadnavirus* X proteins in inhibiting the IFN-β signaling pathway. Further investigations are required to understand the evasion strategy of viruses belonging to *Orthohepadnavirus*.

## 4. Materials and Methods

### 4.1. Plasmids

IFN-Beta_pGL3 was a gift from Nicolas Manel (Addgene plasmid # 102597; http://n2t.net/addgene:102597; accessed on 21 February 2024; RRID:Addgene_102597) [52]. pEF-Bos TRIF Flag was a gift from Kate Fitzgerald and Tom Maniatis (Addgene plasmid # 41550; http://n2t.net/addgene:41550; accessed on 21 February 2024; RRID:Addgene_41550) [53]. pMP31-1 (MAVS plasmid) was a gift from Harmit Malik (Addgene plasmid # 45905; http://n2t.net/addgene:45905; accessed on 21 February 2024; RRID:Addgene_45905) [54]. IgK-IFN-luc was a gift from David Baltimore (Addgene plasmid # 14886; http://n2t.net/addgene:14886; accessed on 21 February 2024; RRID:Addgene_14886) [55]. pcDNA3 IKKe Flag was a gift from Tom Maniatis (Addgene plasmid # 26201; http://n2t.net/addgene:26201; accessed on 21 February 2024; RRID:Addgene_26201) [53]. Human V5-IRF3-pcDNA3 was a gift from Saumen Sarkar (Addgene plasmid # 32713; http://n2t.net/addgene:32713; accessed on 21 February 2024; RRID:Addgene_32713) [56]. pRL-TK (Promega, Madison, WI, USA, Cat# E2241) and pGL4.45[luc2P/ISRE/Hygro] (Promega, Cat# E4141) are commercially available.

cDNA sequences of 17 *Orthohepadnavirus* X proteins with an N-terminal HA-tag were synthesized with codon optimization to human cells (Twist Bioscience, San Francisco, CA, USA). Synthesized DNA sequences are summarized in Supplementary Table S1. Inserts encoding cDNA were cloned into the pCAGGS vector [57], predigested with EcoRI-HF (New England Biolabs [NEB], Ipswich, MA, USA, Cat# R3101M) and NheI-HF (NEB, Cat# R3131M) using In-Fusion Snap Assembly Master Mix (TaKaRa, Kusatsu, Japan, Cat# Z8947N). Plasmids were amplified using NEB 5-alpha F Iq Competent *E. coli* (High Efficiency) (NEB, Cat# C2992H) and extracted with PureYield Plasmid Miniprep System (Promega, Cat# A1222). Sequences of all plasmids were verified using SupreDye v3.1 Cycle Sequencing Kit (M&S TechnoSystems, Osaka, Japan, Cat# 063001) with Spectrum Compact CE System (Promega).

### 4.2. Construction of Plasmids Encoding Orthohepadnavirus X Proteins with Deletions

To construct pCAGGS vectors of *Orthohepadnavirus* X proteins with deletions, mutagenesis was performed with overlapping PCR using PrimeSTAR GXL DNA polymerase (TaKaRa, Cat# R050A). Primers are listed in Supplementary Table S2. The PCR protocol consisted of 35 cycles at 98 °C for 10 s, 60 °C for 15 s, and 68 °C for 1 min, followed by 68 °C for 7 min. Amplified PCR fragments encoding the deletion were cloned into the pCAGGS vector, as described in Section 4.1. Plasmids were verified by sequencing.

### 4.3. Construction of Plasmids Encoding Myc-Tagged TRIF

To construct the pCAGGS vector encoding Myc-tagged human TRIF, the insert encoding human TRIF was PCR amplified from the pEF-Bos TRIF Flag plasmid using PrimeSTAR GXL DNA polymerase. The primers are listed in Supplementary Table S3. The PCR protocol consisted of 35 cycles at 98 °C for 10 s, 60 °C for 15 s, and 68 °C for 1 min, followed by 68 °C for 7 min. Amplified PCR fragments encoding human TRIF were cloned into the pCAGGS vector, as described in Section 4.1. Plasmids were verified by sequencing.

### 4.4. Construction of Plasmids Encoding Chimeric DCH X Proteins

To construct pCAGGS vectors encoding chimeric X proteins, mutagenesis was performed with overlapping PCR using PrimeSTAR GXL DNA polymerase. Primers are listed in Supplementary Table S4. The PCR protocol consisted of 35 cycles of 98 °C for 10 s, 60 °C for 15 s, and 68 °C for 1 min, followed by 68 °C for 7 min. Amplified PCR fragments encoding DCH (Sydney) and DCH (TR-SV15) X proteins were mixed and cloned into the pCAGGS vector using NEBuilder HiFi DNA Assembly Master Mix (NEB, Cat# E2621F). Plasmids were amplified as described in Section 4.1. Plasmids were verified by sequencing.

### 4.5. Cell Culture

Lenti-X 293T cells (TaKaRa, Cat# Z2180N) were cultured in Dulbecco’s modified Eagle’s medium (DMEM) (Nacalai Tesque, Kyoto, Japan, Cat# 08458-16) supplemented with 10% fetal bovine serum (Cytiva, Shinjuku-Ku, Japan, Cat# SH30396) and 1 × penicillin–streptomycin (Nacalai Tesque, Cat# 09367-34).

### 4.6. Generation of 293T-ISRE-luc2 Cells

Briefly, 293T cells (ATCC, Cat# CRL-3216) were transfected with 1 µg pGL4.45[luc2P/ISRE/Hygro] Vector with TransIT-LT1 Transfection Reagent (TaKaRa, Cat# V2304T) in Opti-MEM (Thermo Fisher Scientific, Minoto-Ku, Japan, Cat# 31985062). After 3 d, the cells were cultured in 250 µg/mL of hygromycin B (Nacalai Tesque, Cat# 09287-84) for 10 d. Single-cell cloning was then performed. After cell growth, we evaluated the induction of luciferase activity upon treatment with recombinant human IFN-β (PeproTech, Cranbury, NJ, USA, Cat# 300-02BC) in each clone.

### 4.7. Western Blotting

To check the expression level of *Orthohepadnavirus* X proteins, pellets of Lenti-X 293T and 293T-ISRE-luc2 cells transfected with 500 ng pCAGGS plasmids encoding HA-tagged X protein were lysed with 2 × Bolt LDS sample buffer (Thermo Fisher Scientific, Cat# B0008) containing 2% β-mercaptoethanol (Bio-Rad, Hercules, CA, USA, Cat# 1610710) and incubated at 70 °C for 10 min. Expression of HA-tagged X proteins was evaluated using Simple Western Abby (ProteinSimple, San Jose, CA, USA) with an anti-HA-Tag (6E2) mouse monoclonal antibody (CST, Danvers, MA, USA, Cat# 2367S, ×200) and Anti-Mouse Detection Module (ProteinSimple, Cat# DM-002). The amount of input protein was measured using Total Protein Detection Module (ProteinSimple, Cat# DM-TP01).

### 4.8. Luciferase Reporter Assay

#### 4.8.1. IFN-β Luciferase Reporter Assay

Lenti-X 293T cells were seeded in a 96-well plate (Fujifilm, Osaka, Japan, Cat# 635-28511) at 3 × 10^4^ cells per well, cultured overnight, and transfected with 2.5 ng pIFN-β-Luc plasmid, 45 ng pRL-TK, 2.5 ng TRIF or MAVS plasmid, and 50 ng pCAGGS plasmid, encoding HA-tagged X protein or pCAGGS empty plasmid, using TransIT-LT1 Transfection Reagent in Opti-MEM. After 24 h, the cells were assayed for luciferase activity with Dual-Glo Luciferase Assay System (Promega, Cat# E2920). Firefly luciferase activity was normalized based on Renilla luciferase activity. Percent relative activity was calculated by comparing normalized luciferase data of *Orthohepadnavirus* X proteins plasmid transfected cells and empty plasmid transfected cells. The assays were repeated at least three times. The data shows mean values ± SD from one representative experiment.

#### 4.8.2. IgK-IFN and ISRE Luciferase Reporter Assay

Lenti-X 293T cells were seeded in a 96-well at 3 × 10^4^ cells per well. After overnight incubation, cells were transfected with 5 ng IgK-IFN-Luc plasmid or ISRE Luc plasmid, 40 ng pRL-TK, 5 ng MAVS or IKKe or IRF-3 plasmid, and 50 ng plasmids encoding *Orthohepadnavirus* X proteins or empty plasmid. At 24 h after transfection, cells were assayed for luciferase activity with a Dual-Glo Luciferase Assay System, as described above.

#### 4.8.3. IFN-β Bioassay in 293T-ISRE-luc2 Cells

The 293T-ISRE-luc2 cells were seeded in a 96-well at 3 × 10^4^ cells per well, cultured overnight, and transfected with 50 ng pRL-TK and 50 ng pCAGGS plasmid encoding *Orthohepadnavirus* X proteins, chimeric DCH X protein, or empty plasmid. At 24 h after transfection, the cells were treated with 50 or 10 U/mL recombinant human IFN-β. At 48 h after transfection, the cells were assayed for luciferase activity with Dual-Glo Luciferase Assay System.

### 4.9. TRIF-Degradation Assay

To investigate the effect of protein X-mediated degradation of TRIF, Lenti-X 293T cells were seeded in a 24-well plate (Fujifilm, Cat# 630-28441) at 1.25 × 10^5^ cells per well. The cells were cultured overnight and co-transfected with 250 ng of pCAGGS plasmid encoding HA-tagged protein X and 250 ng of pCAGGS plasmid encoding Myc-tagged human TRIF. Cellular lysates were prepared as described above. The expression of Myc-tagged TRIF was measured using an anti-Myc (9B11) mouse monoclonal antibody (CST, Cat# 2276S, ×100) and Anti-Mouse Detection Module. The amount of input protein was measured using Total Protein Detection Module, as described above.

### 4.10. Immunofluorescence Assay

Lenti-X 293T cells were plated on collagen-coated, 24-well plates (IWAKI, Yoshida, Japan, Cat# 4860-010), cultured overnight, and transfected with 500 ng of pCAGGS vector encoding *Orthohepadnavirus* protein X or pCAGGS empty plasmid using TransIT-LT1 Transfection Reagent. At 24 h after transfection, the cells were fixed in 3% paraformaldehyde (Fujifilm, Cat# 163-20145) and permeabilized with 0.2% Triton X-100 (Sigma Aldrich, Meguro-Ku, Japan, Cat# 9002-93-1). HA-tagged *Orthohepadnavirus* protein X was probed with Alexa Flour 647 anti-HA.11, mouse IgG1 antibody (BioLegend, Cat# 682404, ×200, San Diego, CA, USA). Nuclei were detected by staining with NucBlue Live ReadyProbes Reagent (Thermo Fisher Scientific, Cat# R37605). The localization of *Orthohepadnavirus* protein X was analyzed with EVOS M7000 Imaging System (Thermo Fisher Scientific).

### 4.11. Alignment of Orthohepadnavirus Protein X and Phylogenetic Analysis

The amino acid sequences of protein X from 17 *Orthohepadnavirus* sp. were aligned using the MUSCLE algorithm in MEGA X (MEGA Software) version 11.0.13. A phylogenetic tree was constructed using the alignment of amino acid sequences from public databases, and evolutionary analysis was conducted using the maximum likelihood method and neighbor-joining method based on the Jones–Taylor–Thornton matrix-based model with 1000 bootstrap replicates.

### 4.12. Statistical Analysis

The results are presented as the mean and standard deviation of four measurements from one assay, representing at least two or three independent experiments. Differences in relative values between *Orthohepadnavirus* X proteins and empty plasmid were examined by one-way ANOVA followed by Dunnett’s multiple comparison test. A *p* ≤ 0.05 was considered statistically significant. Analysis was performed using Prism 10 software v10.1.2 for Windows (GraphPad Software, Boston, MA, USA).

## Data Availability

Data is contained within the article and Supplementary Materials.

## Supplementary Materials

The supporting information can be downloaded at: https://www.mdpi.com/article/10.3390/ijms25073753/s1.
