# Supplementary material for: Conserved Functions of Orthohepadnavirus X Proteins to Inhibit Type-I Interferon Signaling
Source: Int J Mol Sci. 2024 Mar 28;25(7):3753. doi: 10.3390/ijms25073753 (PMC11011558; doi:10.3390/ijms25073753)
Supplement: Supplementary file 1 [file ijms-25-03753-s001.zip › 240221 Supplementary Table Final.pdf]

Supplementary Table S1. Synthesized DNAs for generating plasmids encoding X protein

X protein	Codon-optimized DNA sequence
HBV (genotype A)<br>Accession #<br>LC488828.1	ATGTACCCTTACGATGTACCTGACTACGCGACCGGTGCCGCTCGACTT<br>TATTGCCAACTTGATCCATCCCGCGACGTGTTGTGTCTCAGACCCGTT<br>GGCGCCGAATCTCGAGGTCGCCCACTTTCAGGACCCCTTGGTACATTG<br>AGTTCTCCATCTCCGAGCGCTGTACCGGCGGACCACGGGGCTCATTTG<br>AGTTTGAGGGGTCTCCCAGTGTGTGCTTTCAGCAGTGCCGGCCCGTGC<br>GCGCTTCGATTCACTTCAGCAAGATGTATGGCTACGACTGTCAATGCT<br>CATCAAATCCTCCCAAAGGTCCTCCACAAAAGAACGCTTGGGCTTCC<br>GGCCATGTCTACTACTGATCTTGAGGCGTACTTCAAGGACTGCGTCTT<br>TAAGGATTGGGAGGAATTGGGGGAGGAAATTCGCCTTAAAGTATTCG<br>TGCTGGGGGGTTGCAGACACAAACTTGTATGCGCTCCTGCCCCGTGC<br>AACTTCTTCACTTCCGCATAA
HBV (genotype D)<br>Accession #<br>YP_009173867.1	ATGTACCCTTACGATGTACCTGACTACGCGACCGGTGCCGCACGCCTC<br>TGTTGCCAACTTGATCCAGCACGGGATGTTCTGTGCCTTCGGCCAGTC<br>GGCGCTGAAAGTTGTGGACGGCCGTTCTCCGGCTCCCTCGGAACGCT<br>CTCCTCCCCCTCCCCCTCAGCCGTCCCTACAGATCATGGAGCTCACCT<br>TTCTCTTCGCGGGCTTCCGGTATGCGCGTTTTCTTCTGCTGGACCTTGC<br>GCGTTGCGCTTTACATCTGCTAGGAGAATGGAAACAACGGTTAACGC<br>GCACCAGATACTCCCGAAGGTTTTGCACAAAAGGACCCTGGGTCTTA<br>GTGCCATGAGCACTACTGATCTCGAGGCATACTTTAAGGACTGCTTGT<br>TCAAGGATTGGGAGGAACTTGGGGAAGAGATCAGATTGAAGGTGTTC<br>GTCTTGGGGGGATGTCGGCATAAGCTCGTGTGTGCACCTGCACCCTG<br>CAATTTCTTCACGTCTGCTTAG
HBV (genotype G)<br>Accession #<br>BAD91282.1	ATGTACCCTTACGATGTACCTGACTACGCGACCGGTGCAGCCCGGCT<br>CTGTTGTCAGTTGGATCCCAGCCGAGACGTTCTCTGCTTGCGGCCAGT<br>TAGCGCAGAGTCATCTGGACGCCCGCTGCCCGGACCTTTTGGTGCACT<br>CTCTCCTCCAAGTCCCTCTGCAGTCCCCGCAGATCATGGCGCTCACCT<br>GTCATTGCGCGGTTTGCCAGTATGCGCCTTCTCCTCAGCAGGTCCGTG<br>CGCGCTTCGATTCACGTCAGCACGGTACATGGAAACGGCAATGAATA<br>CGAGCCACCATCTCCCCCGACAACTTTACAAACGCACCCTCGGTCTTT<br>TCGTTATGAGTACCACTGGTGTCGAGAAGTACTTTAAAGACTGTGTCT<br>TCGCTGAATGGGAGGAGTTGGGCAACGAGAGCCGACTGATGACTTTC<br>GTGCTGGGCGGATGCAGACATAAGCTCGTGTGTGCTCCGGCCCCCTG<br>TAATTTCTTTACTTCAGCTTAA
HBV (genotype H)<br>Accession #<br>BAJ15483.1	ATGTACCCTTACGATGTACCTGACTACGCGACCGGTGCCGCTAGGCT<br>GTGCTGCCAACTTGATCCCGCCCGCGACGTGCTGTGTTTGCGGCCAGT<br>AGGTGCTGAAAGCTGCGGTCGGCCACTTTCCTGGTCCCTCGGGGCTTT<br>GCCCCCGTCATCACCTCCAACAGTTCCTGCTGACGACGGATCTCACTT<br>GAGTCTTCGGGGGTTGCCCGCGTGCGCGTTCAGTTCCGCTGGTCCTTG<br>TGCGTTGCGATTTACGAGTGCTAGGAGAATGGAAACGACTGTCAACG<br>CTCCCTGGAACTTGCCCACTACGCTGCACAAACGAACATTGGGTCTGT<br>CTCCCCGCTCCACGACTTGGATAGAAGAGTACATTAAAGACTGTGTTT<br>TCAAAGACTGGGAGGAAAGTGGGGAGGAACTTCGCCTGAAGGTGTTT<br>GTGCTTGGCGGTTGTAGACACAAACTCGTGTGTTCCCCGGCCCCTTGT<br>AACTTCTTCACTAGCGCATAG

DCH (KT-116)<br>Accession #<br>LC668427.1	ATGTACCCTTACGATGTACCTGACTACGCGACCGGTGCAGCACGGCT<br>GCGCTGCGAACTCGATCCTTCTGGTCGGGTCCTGCGGTTGAGACCATT<br>CATTAGTGAATCCAGCGGACGCGCGGTTAGCCGAACTGCACGCTTGC<br>CAGACCTGAGCCCCTCAGTTGCGGTTTCAGCGACACTGCGGGCCAGG<br>GAATCTCTGAGGGGTATACCTGCCTGTCTCACGTCACCAGAGGGCCC<br>TTGTGTTTTGAGATTTACCTGCGCTGATAGTAGACGGTGCATGGAAGC<br>AGCAATGATTGGCTTGGTCCCAGCACTGCTTGCTCGCCAACTTGGCTT<br>CGGGACTTGGCAGCCGGATGTATGGACGCTTCGGCTTCGCGATCTTTT<br>GTTGGTCGAGTGGGAGGAAGAAGGACTGACGCCGCGGTTGTGTACTT<br>ATCTTGTAACGGGGTGCGCTCATAAAACGCTTCACACTCGATAG
DCH (Rara)<br>Accession #<br>LC685967.1	ATGTACCCTTACGATGTACCTGACTACGCGACCGGTGCAGCCCGCCTC<br>AGATGTGATCTGGACTCATCTGGGCGAGTTCTTCGACTTCGCCCGTTC<br>CATTCCGAGTCCTGTGGCAGGGCCGTCAGCAGGGCTTCTAGGTTGCC<br>AGACGTGCCCACTGCCGTTGCGGTGCCGAGTACACTTCGACCCTGTC<br>AGTCTTTGCGAGGATTGCCAGCATGTCTCGCATCAAGCGCTGGCCCCT<br>GTGTGCTCCGGTTTACTTGCGCCGACTGTAGGAGGTGTATGGAGACA<br>GCCATGATCGGTCTTGTCCCCGCGCTTTTGGCCAGGCAACTTGGGAGT<br>GGGACGTGGCAAACTGATCTTTGGACACTCCGACTTCGGGAGTTGCT<br>GCTTGCTGAATGGGAGGAGGAGGGACTTACACCGAGACTCTGTACTT<br>ACCTTCTCTCTGGCTGTGCACATAAGACTCTCTATCCATGCTGA
DCH (Sydney)<br>Accession #<br>MH307930.1	ATGTACCCTTACGATGTACCTGACTACGCGACCGGTGCGGCTCGGCTT<br>AGGTGCGAGCTTGACCCGTGGGGAAGGGTGTTGAGGCTGAGACCTTT<br>TCATTCAGAGTCCGGTGGAAGGGCGGTATCTAGGACTGCTCGACTCC<br>CGGATCTTAGTACGTCCGTGGCTGTGTCCGCCACTCTCAGAGCCAGG<br>GAATCTCTTCGAGGCATCCCTGCCTGTCTTACTAGTCCCGAAGGGCCG<br>TGTGTTCTTCGGTTCACCTGCGCTGACTCCCGACGATGTATGGAGGCG<br>GCAATGACTGGTCTCGTACCCGCTCTCCTTGCCAGACAACTGGGATTT<br>GGGACCTGGCAACCCGATCTTTGGACCTTGCGGCTTCGGGACCTCCTG<br>CTTGTAGAGTGGGAGGAAGAGGGGTTGACACCCAGGCTTTGCACTTA<br>CCTGGTGACAGGATGTGCTCATAAGACACTTCATACGAGGTGA
DCH (TR-SV15)<br>Accession #<br>ON293153.1	ATGTACCCTTACGATGTACCTGACTACGCGACCGGTGCTGCTAGATTG<br>AGATGTGAGTTGGATCCCTCTGGACGAGTGCTGCGGTTGAGACCATT<br>CCATTCTGAGTCTTCCGGGCGGGCTGTCAGCAGAACTGCGCGGTTGC<br>CGGACCTCTCAACTAGTGTAGCGGTAAGTGCTACACTCCGCGCAAGA<br>GAGTCACTGCGAGGCATCCCAGCTTGTCTGACCTCTCCAGAAGGTCCT<br>TGCGTTCTCAGATTCACATGCGCCGATTCTCGGCGGTGTATGGAGGCA<br>GCAATGATAGGTCTTGTCCCAGCATTGCTGGCCAGGCAACTCGGATTT<br>GGCATATGGCAACCTGAGCTTTGGACTCTTAGATTGCGCGACCTCCTC<br>CTGGTCGAATGGGAAGAAGAAGGTCTGACTCCAAGACTGTGTACATA<br>T CTGGTTACTGGCAGCGCTCACCAGACCCTGCACAATCGGTGA
WHV<br>Accession #<br>NC_004107.1	ATGTACCCTTACGATGTACCTGACTACGCGACCGGTGCTGCTAGGCTC<br>TGCTGCCAGCTCGATTCTGCCCGAGACGTGTTGCTGCTTCGACCGTTT<br>GGACCTCAAAGCAGTGGACCTAGTTTTCCGAGACCGGCGGCAGGTTC<br>AGCAGCCAGCAGCGCTTCATCACCGAGTCCGAGTGACGAATCAGATT<br>TGCCGCTCGGCAGACTCCCTGCATGTTTTGCTAGTGCGTCTGGACCCT<br>GCTGCCTGGTTTTTACTTGTGCAGAATTGAGAACCATGGATTCCACGG<br>TAAACTTCGTATCTTGGCATGCCAACCGGCAGCTGGGTATGCCGTCCA<br>AAGATTTGTGGACGCCCTATATAAAGGATCAGCTCCTCACCAAATGG

	GAAGAAGGCTCTATCGACCCACGCCTGAGTATCTTCGTGTTGGGGGG<br>TTGTCGCCATAAATGTATGCGATTGCTCTAA
Domestic donkey<br>HBV<br>Accession #<br>QMV34684.1	ATGTACCCTTACGATGTACCTGACTACGCGACCGGTGCGGCTCGCCTG<br>AGGTGTCAACTGGACCCAACTGGCCGGGTACTCCACCTCCGACCCTTT<br>ACTTCCGAATCTTGTAGAAGAACTTTGGCAGGTACTGCCGGGGCACC<br>AGATCTCCCAGCAGCGGACCTCCTTCAAGCGGATCACCGGACTCATC<br>TTAGGGTTCGACGCTTGCCTGCTTGCTGTTTCTCTTCTCGCGGTCCGTG<br>TGTGCTTAGGTTCACATGCGCGGACCTTAGCCGACGAATGGAAGCCC<br>CGATGAACCTCGTTCAATATCTGGGGAAAAGGGCGCGGGGTCTTCAG<br>CATCCGCCCGGTGATTCCTATTGCCAACATGAACTTTGGACACAATGG<br>GAGGAGAATGGTTGGTCAGACAGAATCTATACTTACGTGTTGGGAGG<br>ATGCAGACACAAATGGCTTTACCCACTTTAG
Asian grey shrew<br>HBV<br>Accession #<br>YP_010796421.1	ATGTACCCTTACGATGTACCTGACTACGCGACCGGTGCTGCAAGAAT<br>GCTCTTCGATCTCGACCCTGCTACAGGAGCTGTACGCCTTCGCCCATT<br>TCTCACTGAACCCCGCGGACGAGGGGAACAGACACCGCGCCCGACTT<br>CCTCTCCGACAACGTCAGCCCTGTCTTCTTTCCTTGGAAGTCGGTCTT<br>CCTGGCGGCGCTTGCCAAGCTGCGCCGACTCTCCATTCGGCCCATGTA<br>CTTTGCGGTTCACGTTCGCAGAGCTGGGAAACTTGCAGACACCAATG<br>AACTCAGTGACCTTCATCAGTTGTCGGTCAAGGGGAGCCCATCTGAA<br>GTGCCGGAGGCAACAGAAGAATTGGACCTGGTATTTCTGGACACATC<br>ATAATGCGAACAACACGCACCATTTGTGGCTTATGTGCTACGGAGGT<br>TGTAGGCATAAATAG
Capuchin monkey<br>HBV<br>Accession #<br>YP_009666527.1	ATGTACCCTTACGATGTACCTGACTACGCGACCGGTGCAGCCAGACT<br>TTGTTGCCAACTGGACCCTGCCAGGGATGTTCTTTGTCTCCGACCTGT<br>AACTGCCGAGCCATGTGGACGACCCTTCAGCGGTTCTGCTCGGACAT<br>CCGCTCCGGCAGCTGCGGCAGCCCTGCCCTCTATTGATGGAGCATATC<br>TGTCCCTTCGAGGGCTTCCTAGTTGCGCTTTCTCATCCTCAGGGCCCT<br>GCGCCTTGAGGTTTACAAGTGCGCGACGAATGGCTACACCGATGAAT<br>AGTAGAGATCTGGTCCAACAACTCTATAATCGGACGTTGGGTCTTGCT<br>CCTCTCTCCACTGGGCAGTGGGAACGGCACTTTAAAGATCTTTTGTTC<br>GAGGAATGGGAGGAACTCGGTGTTGAGTTCAGGTTGAAAGTATTCGT<br>GCTGGGGGGTTGTCGCCATAAGCTCGTTTGCAGTGTGCAACCTTGCAT<br>ATTCTTCACTAGTGCCTAA
Woolly monkey HBV<br>Accession #<br>YP_009175037.1	ATGTACCCTTACGATGTACCTGACTACGCGACCGGTGCAGCAAGACT<br>GTGTTGCTACTTGGACCCTGAAAGAGATGTACTCTGTCTTCGCCCTCT<br>CCAAGCAGAACCCTCCGGCCGGCCATTTTCTGGATTGTCTAGACCAG<br>CAGAGACGGCTGCGGCAGCGGCCGTTCCAGCCTTCCACGGTGCACAT<br>CTGTCTCTGAGAGGTCTTCCCAGTTGTGCGTTCTCATCAGCCGGCCCA<br>TGTGCCTTGAGGTTTACATCTGCAACGTGGAGATGCATGGAAACCCC<br>AATGAATTCTGTGACCTGCTTGCGAAAGAAGACTCTGGGACTGCGGA<br>CTGCACCACCAACCGTGATGGAACAATACATTAAGGACTGTTTGTTT<br>GAACAATGGGAGGAGCAAGGTGAGGAACCACGACTGAAAGTATTC<br>GTCCTTGGTGGGTGTCGGCACAAGCTCGTGGGGACGGCCTCACCGTG<br>CATCTTTTTCACTTCCGCATAG
Orangutan HBV	ATGTACCCTTACGATGTACCTGACTACGCGACCGGTGCTGCCCGCCTT<br>TGTTGTCAGTTGGACCCGGCTCGAGATGTCCTTTGCCTTCGGCCAGTG<br>GGAGCTGAGAGTAGAGGAAGGCCGTTCCCAGGCAGTATTGGTGCTCT<br>GCCCCCACCATCTCTGAGCGCGGTACCGGCCGACCACGGAGCCCACC

Accession #<br>AAF33122.1	TTAGCCTTCGGGGTTTGCCGGTATGCGCTTTTTCTTCAGCGGGGCCTT<br>GTGCGTTGAGGTTCACGAGCGCCAGGTGCATGGAGACCACCGTAAAT<br>GCGCCTAGAAATCTCCCTAAGGTCCTGCATAAGAGAACATTGGGCCT<br>TTCCACTATGTCTACTACGCGAATCGAAACGTACTTTAAGGATTGTGT<br>GTTTAAGGATTGGGAAGAACTTGGAGAGGAGATCCGGTTGAAGGTTT<br>TTGTCTTGGGTGGATGTAGGCATAAATTGGTGTGTTCTCCCGCGCCTT<br>GCAACTTTTTTACAAGTGCATGA
Horseshoe bat HBV<br>Accession #<br>YP_009045997.1	ATGTACCCTTACGATGTACCTGACTACGCGACCGGTGCCGCCCGCCTG<br>CACTGTGAACTGGATGCTGCGAGGGATGTGCTGTTGCTGAGACCACT<br>TGGCACTCAGCCCGGAGGGCGCAGTGTCGCTAGGGCGGCTAGAGATC<br>CAGCCGGGGCTGCGGCTGCTGCAGTTCCCAGCGTCCATAGACCCCAT<br>TTGCCAGTCAGAAGGTTGCCTGCGTGTGCCTTTACGCCCGCCGGCCCC<br>TGTGTCCTCCGCTTCACTTGTGCAGATCTTCAAAGACACATGGAGACG<br>ACCATGAATTTCGTACCGTGGCAGATGGCACGCCAACGGGGGCAGCT<br>CATGAGAACTCTCTCATACTGGGATTGGTATTTCAAGCAATCCTTGAT<br>GAACCAGTGGGAGGAGCAAGGCCTTGGTGAGAGATTGAACACCTAC<br>GTGCTCGGAGGATGTAGGCATAAGTTGCGATGA
Tent-making bat HBV<br>Accession #<br>NC_024445.1	ATGTACCCTTACGATGTACCTGACTACGCGACCGGTGCAGCACGGAT<br>GCTTTGCCACTATGACACCGCCCGCGATATTCTGCATCTTAGACCCCT<br>TAGAGGCCAAAGTTCTGGCCCGGCACTCGCCGGGACACCCGCCGCAC<br>AAGCAGCCAGTCCGCCCCCCCCGGTTTCCCCAGATCATAGACCTGAT<br>CTCTCACTCAGAGCTTTGCCAGCTTGTGCAACATCAGACGCAGGTCCA<br>TGTGTATTGAGATTTACCTCCGCGGATCTCAGGCGGCTTGCTACGCCA<br>GTAAACATCTGTCATAGCCTGACCAGACGCACCAGGGGGCATCCATG<br>GACACTGAACAATATGGACTCATGGACATGGCTGATGATTAACCGCG<br>AGGTTGGTCCACGCATTGAGTATGTTCTTGTCGCCGGCGGCTGCAGAC<br>ACAAACTGCAGTAA
Pomona bat HBV<br>Accession #<br>YP_009506648.1	ATGTACCCTTACGATGTACCTGACTACGCGACCGGTGCGGCCCGGTT<br>GTACTGTGAACTGGACCCCTCTCGGGACGTATTGCTCCTGAGACCGTT<br>TGGCGCACAACCAGGAGGGCGGCCAGTCAGTCGGTTTCCTGGCGATT<br>CAAATGGTTCTACCGCATCTACCGTGCCACCCGTACACAGACAGGAT<br>TTCGCCTTGAGGAGGCTCCCGGCTTGTGCTTACAGTAACGCAGGTCCG<br>TGTGTGCTTCGGTTCACTTGTGCCGAAGCTGCAGTAAACATGGAGAC<br>AACGATGACCAACTTTGTAACTTGGCATACAGCCCGCCAGCGCGGGA<br>CTGTAACCCAGACCCTCAATTCCTGGCAGTGGTATTTTGGTCAACTGT<br>TGATGAACCAATGGGAAGAGACCGGTTGGGACGATCGCATTATCGCG<br>TAT GTACTTGGCGGTTGTCGACACAAGCTGAGGTGA

Supplementary Table S2. Primers used for generating plasmids encoding deletion mutant X proteins of HBV(A) and DCH (KT-116)

X protein	Deletion region	Direction	Sequence (5′-3′)
HBV (genotype A) Accession # LC488828.1	52-148	Forward	TGACTACGCGACCGGTGCCGCTCGA
		Reverse	AAAAAGATCTGCTAGCTTATGCGGAAGTGAAGAAGTTAGCCCCGTGGTCCGCCGGTACAGCG
	120-140	Forward	TGACTACGCGACCGGTGCCGCTCGA
		Reverse (120-140R)	ATCCTTAAAGACGCAGTCCT
		Forward (120-140F)	TGCGTCTTTAAGGATCTTGTATGCGCTCCTGCCCC
		Reverse	AAAAAGATCTGCTAGCTTATGCGGA
	1-50	Forward	TGACTACGCGACCGGTGCTCATTTGAGTTTGAGGGG
		Reverse	AAAAAGATCTGCTAGCTTATGCGGA
	45-140	Forward	TGACTACGCGACCGGTGCCGCTCGA
		Reverse (45-140R)	AGCGCTCGGAGATGGAGAAC
		Forward (45-140F)	CCATCTCCGAGCGCTCTTGTATGCGCTCCTGCCCC
		Reverse	AAAAAGATCTGCTAGCTTATGCGGA
	88-100	Forward	TGACTACGCGACCGGTGCCGCTCGA
		Reverse (88-100R)	TTGATGAGCATTGACAGTCG
		Forward (88-100F)	GTCAATGCTCATCAACCGGCCATGTCTACTACTGA
		Reverse	AAAAAGATCTGCTAGCTTATGCGGA
DCH (KT-116) Accession # LC668427.1	52-145	Forward	TGACTACGCGACCGGTGCAGCACGG
		Reverse	AAAAAGATCTGCTAGCCTAGGCCCGCAGTGTCGCTG
	120-140	Forward	TGACTACGCGACCGGTGCAGCACGG
		Reverse	AAAAAGATCTGCTAGCCTATCGAGTGTGAAGCGTCTCGACCAACAAAAGATCGC

	1-50	Forward	TGACTACGCGACCGGTGCCAGGGAATCTCTGAGGGG
		Reverse	AAAAAGATCTGCTAGCCTATCGAGT
	45-140	Forward	TGACTACGCGACCGGTGCAGCACGG
		Reverse	AAAAAGATCTGCTAGCCTATCGAGTGTGAAGCGTCGCAACTGAGGGGCTCAGGT
	88-100	Forward	TGACTACGCGACCGGTGCAGCACGG
		Reverse (88-100R)	GCCAATCATTGCTGCTTCCA
		Forward (88-100F)	GCAGCAATGATTGGCGGGACTTGGCAGCCGGATGT
		Reverse	AAAAAGATCTGCTAGCCTATCGAGT

Supplementary Table S3. Primers used for generating a plasmid encoding Myc-tagged TRIF protein

Direction	Sequence (5′-3′)
Forward	TTTTGGCAAAGAATTCGCCACCATGGCCTGCACAGGCCCATCACTTC
Reverse	AAAAAGATCTGCTAGCTTACAAATCTTCTTCGGAGATCAACTTCTGCTCTTCTGCCTCCTGCGTCTTGTC

Supplementary Table S4. Primers used for generating plasmids encoding chimeric DCH X protein

X protein	Chimeric region	Direction	Sequence (5′-3′)
DCH (Sydney) and DCH (TR-SV15)	Sydney (1-100)/ TR-SV15 (101-145)	Forward	TTTTGGCAAAGAATTCGCCACCATG
		Reverse (Sydney 100R)	TGCCATATGCCAAATCCCAGTTGTCTGGCA
		Forward (TR-SV15 100F)	ATTTGGCATATGGCAACCTGAG
		Reverse	AAAAAGATCTGCTAGCTCACC
	TR-SV15 (1-100)/ Sydney (101-145)	Forward	TTTTGGCAAAGAATTCGCCACCATG
		Reverse (TR-SV15 100R)	TGCCAGGTCCCAAATCCGAGTTGCCTGGCC
		Forward (Sydney 100F)	ATTTGGGACCTGGCAACCCGAT
		Reverse	AAAAAGATCTGCTAGCTCACC
