# Supplementary material for: Conserved Functions of Orthohepadnavirus X Proteins to Inhibit Type-I Interferon Signaling
Source: Int J Mol Sci. 2024 Mar 28;25(7):3753. doi: 10.3390/ijms25073753 (PMC11011558; doi:10.3390/ijms25073753)
Supplement: Supplementary file 1 [file ijms-25-03753-s001.zip › ijms-2906135-supplementary.pdf]

International Journal of *Molecular Sciences*

MDPI

A

HBV (A)	100%																
HBV (D)	100%	100%															
HBV (G)	100%	100%	100%														
HBV (H)	100%	100%	100%	100%													
DCH (KT-116)	39.87%	39.87%	39.87%	39.87%	100%												
DCH (Rara)	39.87%	39.87%	39.87%	39.87%	100%	100%											
DCH (Sydney)	39.87%	39.87%	39.87%	39.87%	100%	100%	100%										
DCH (TR-SV15)	39.87%	39.87%	39.87%	39.87%	100%	100%	100%	100%									
WHV	41.71%	41.71%	41.71%	41.71%	45.39%	45.39%	45.39%	45.39%	100%								
DDHBV	42.94%	42.94%	42.94%	42.94%	53.37%	53.37%	53.37%	53.37%	45.39%	100%							
AGSHBV	31.28%	31.28%	31.28%	31.28%	37.42%	37.42%	37.42%	37.42%	36.19%	36.8%	100%						
CMHBV	67.48%	67.48%	67.48%	67.48%	41.71%	41.71%	41.71%	41.71%	44.78%	46.01%	35.58%	100%					
WMHBV	66.25%	66.25%	66.25%	66.25%	45.39%	45.39%	45.39%	45.39%	42.33%	46.01%	35.58%	69.93%	100%				
OHBV	87.73%	87.73%	87.73%	87.73%	39.26%	39.26%	39.26%	39.26%	42.94%	43.55%	30.67%	69.93%	68.09%	100%			
HSBHBV	42.94%	42.94%	42.94%	42.94%	50.92%	50.92%	50.92%	50.92%	53.98%	53.98%	41.71%	47.85%	50.3%	44.17%	100%		
TMBHBV	39.26%	39.26%	39.26%	39.26%	41.1%	41.1%	41.1%	41.1%	46.01%	44.78%	41.71%	37.42%	38.65%	38.03%	49.07%	100%	
PBHBV	44.17%	44.17%	44.17%	44.17%	49.69%	49.69%	49.69%	49.69%	53.37%	50.3%	41.71%	43.55%	46.62%	43.55%	68.71%	47.85%	100%
	HBV (A)	HBV (D)	HBV (G)	HBV (H)	DCH (KT-116)	DCH (Rara)	DCH (Sydney)	DCH (TR-SV15)	WHV	DDHBV	AGSHBV	CMHBV	WMHBV	OHBV	HSBHBV	TMBHBV	PBHBV

B

HBV (A)	100%																
HBV (D)	94.47%	100%															
HBV (G)	94.47%	94.47%	100%														
HBV (H)	94.47%	94.47%	94.47%	100%													
DCH (KT-116)	42.48%	42.48%	42.48%	42.48%	100%												
DCH (Rara)	42.48%	42.48%	42.48%	42.48%	88.95%	100%											
DCH (Sydney)	42.48%	42.48%	42.48%	42.48%	88.95%	88.95%	100%										
DCH (TR-SV15)	42.48%	42.48%	42.48%	42.48%	88.95%	88.95%	88.95%	100%									
WHV	9.96%	9.96%	9.96%	9.96%	10.42%	10.42%	10.42%	10.42%	100%								
DDHBV	11.04%	11.04%	11.04%	11.04%	12.57%	12.57%	12.57%	12.57%	9.81%	100%							
AGSHBV	8.74%	8.74%	8.74%	8.74%	8.89%	8.89%	8.89%	8.89%	8.12%	8.89%	100%						
CMHBV	15.95%	15.95%	15.95%	15.95%	11.04%	11.04%	11.04%	11.04%	10.73%	11.65%	9.5%	100%					
WMHBV	16.25%	16.25%	16.25%	16.25%	11.5%	11.5%	11.5%	11.5%	10.12%	11.5%	9.5%	16.87%	100%				
OHBV	20.85%	20.85%	20.85%	20.85%	10.58%	10.58%	10.58%	10.58%	10.58%	11.19%	8.74%	16.56%	16.71%	100%			
HSBHBV	11.19%	11.19%	11.19%	11.19%	11.34%	11.34%	11.34%	11.34%	11.8%	12.42%	9.35%	12.42%	12.11%	11.34%	100%		
TMBHBV	10.58%	10.58%	10.58%	10.58%	9.2%	9.2%	9.2%	9.2%	9.96%	9.81%	9.2%	10.12%	9.35%	10.42%	10.58%	100%	
PBHBV	11.34%	11.34%	11.34%	11.34%	10.42%	10.42%	10.42%	10.42%	11.8%	11.04%	9.2%	10.88%	11.04%	11.04%	15.03%	9.96%	100%
	HBV (A)	HBV (D)	HBV (G)	HBV (H)	DCH (KT-116)	DCH (Rara)	DCH (Sydney)	DCH (TR-SV15)	WHV	DDHBV	AGSHBV	CMHBV	WMHBV	OHBV	HSBHBV	TMBHBV	PBHBV

Supplementary Figure S1

(A) Sequence identity of X proteins. **(B)** Sequence similarity of X proteins. Sequence identity and similarity were calculated by Sequence Identities and Similarities website (http://imed.med.ucm.es/cgi-bin/sias_new.cgi?jobid=1710329285, accessed on 13 March 2024).

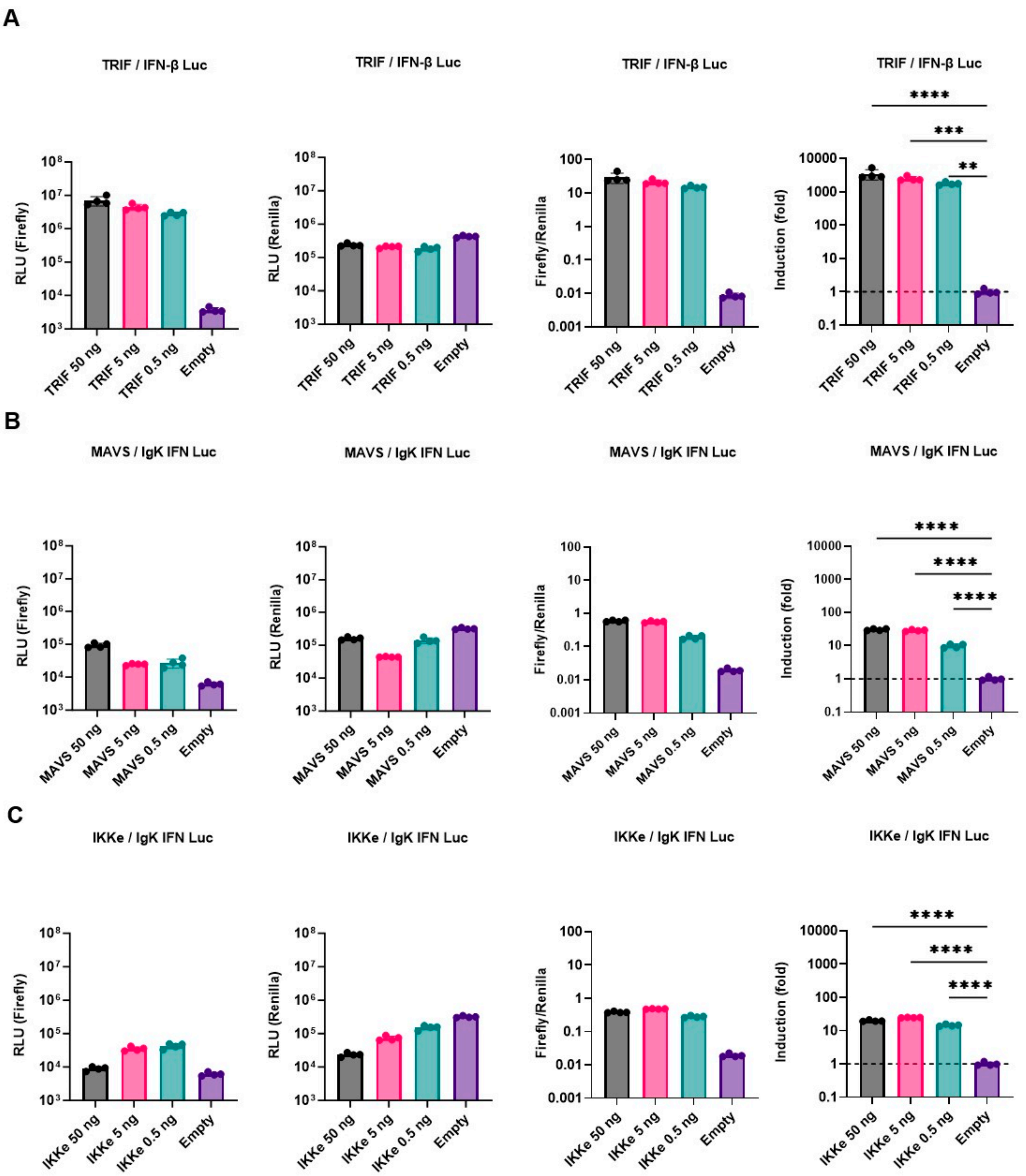
A
TRIF / IFN-β Luc
RLU (Firefly)
RLU (Renilla)
Firefly/Renilla
Induction (fold)
TRIF 50 ng
TRIF 5 ng
TRIF 0.5 ng
Empty
****
***
**
B
MAVS / IgK IFN Luc
MAVS 50 ng
MAVS 5 ng
MAVS 0.5 ng
****
C
IKKe / IgK IFN Luc
IKKe 50 ng
IKKe 5 ng
IKKe 0.5 ng

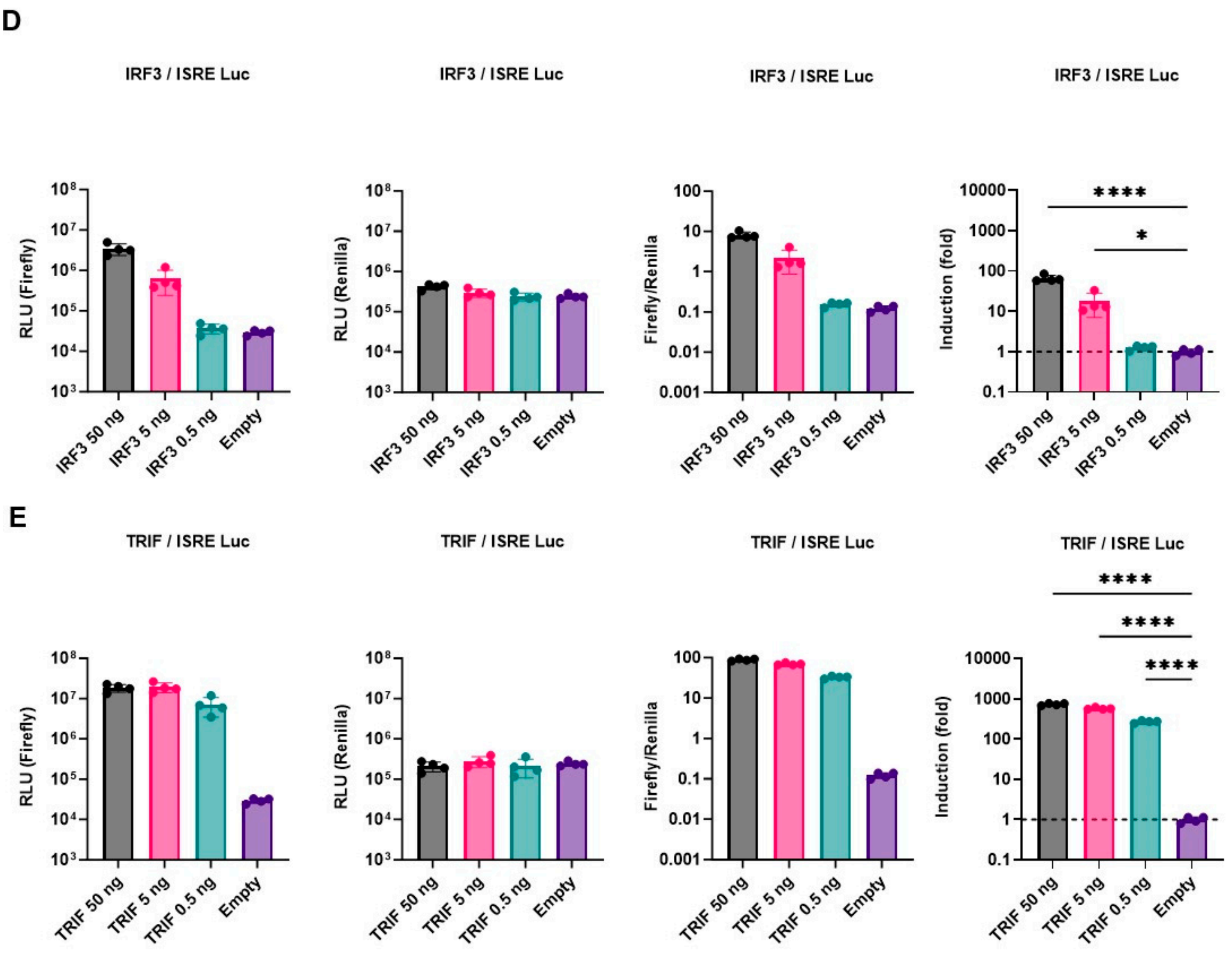

Supplementary Figure S2. Induction of Firefly luciferase.

Co-transfection of the following plasmids **(A)** IFN-β Luc and TRIF, **(B)** IgK-IFN Luc and MAVS, **(C)** IgK-IFN Luc and IKKe, **(D)** interferon-stimulated response element (ISRE) Luc and IRF3, and **(E)** ISRE Luc and TRIF. Differences between IFN-stimulating plasmid and empty plasmid were examined by one-way ANOVA followed by Dunnett's multiple comparison test. ****$p < 0.0001$, ***$p < 0.001$, **$p < 0.01$, and *$p < 0.05$.

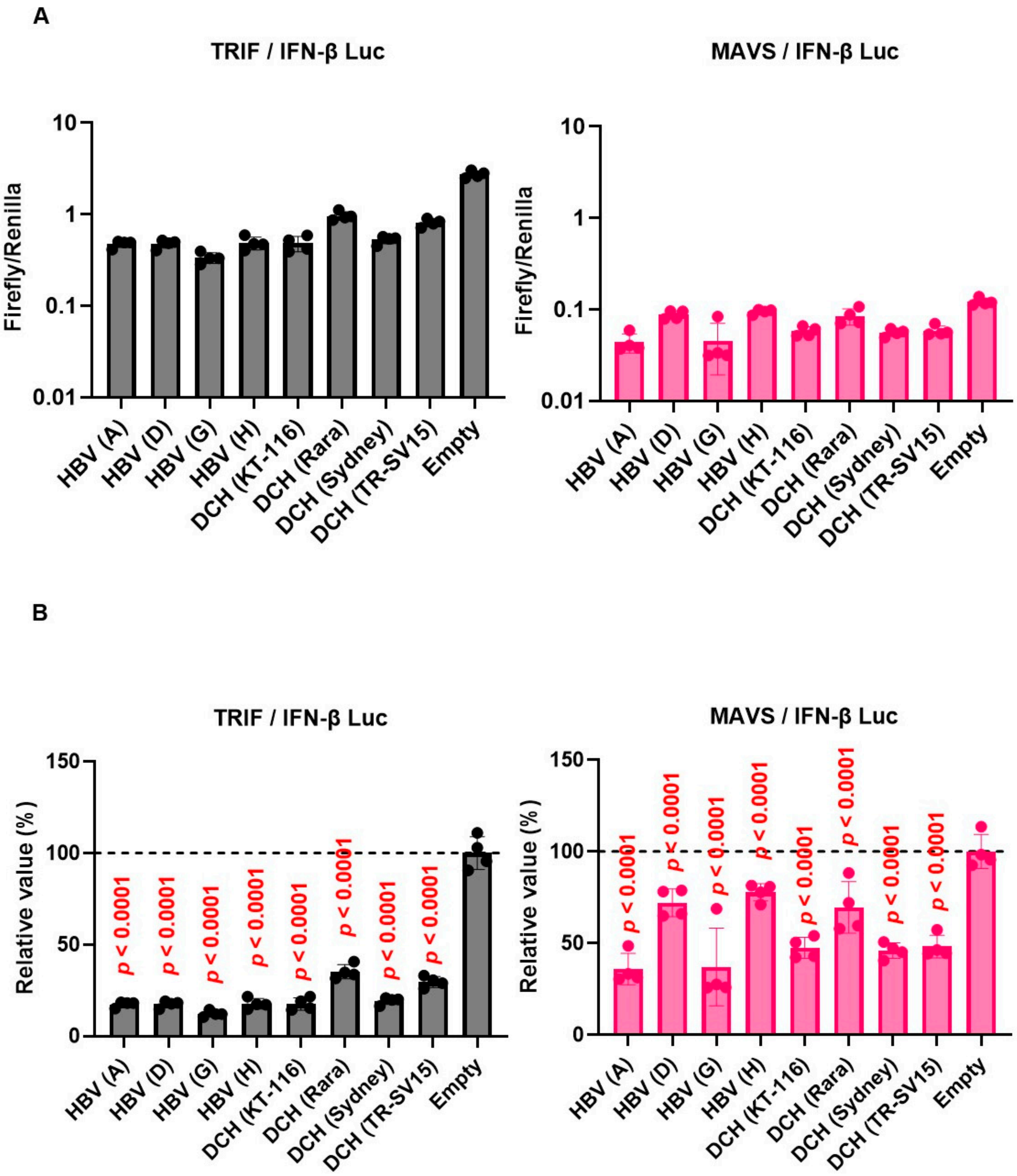

Supplemental Figure S3. ***Orthohepadnavirus*** **X protein inhibits IFN-β induction mediated by TRIF and MAVS. (A)** Raw data of the luciferase reporter assay. The RLU of Firefly luciferase was divided by the RLU of Renilla luciferase. **(B)** Relative value of IFN-β luciferase reporter assay. Differences between *Orthohepadnavirus* X proteins and "Empty" were examined by one-way ANOVA followed by Dunnett's multiple comparison test.

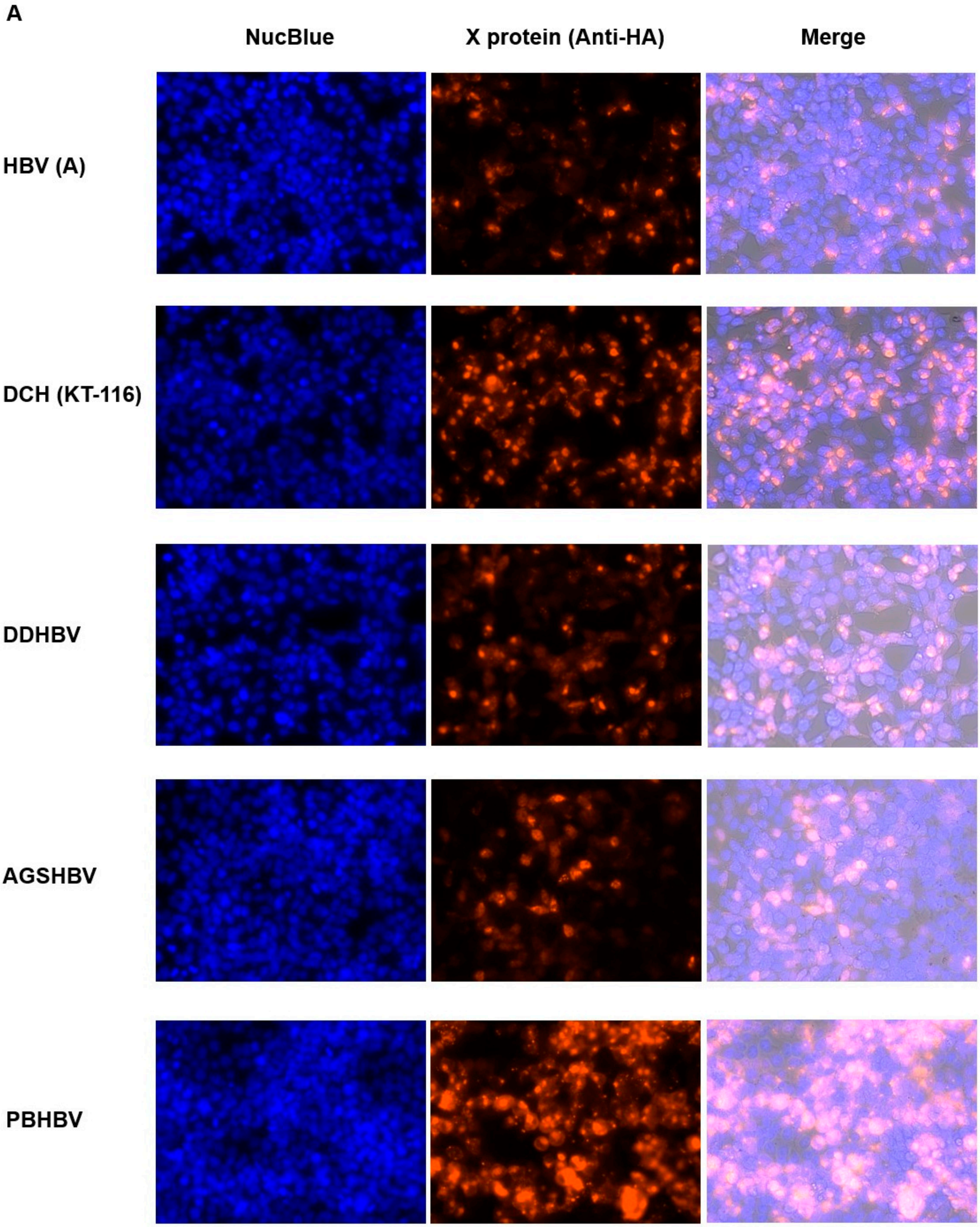
A
NucBlue
X protein (Anti-HA)
Merge
HBV (A)
DCH (KT-116)
DDHBV
AGSHBV
PBHBV

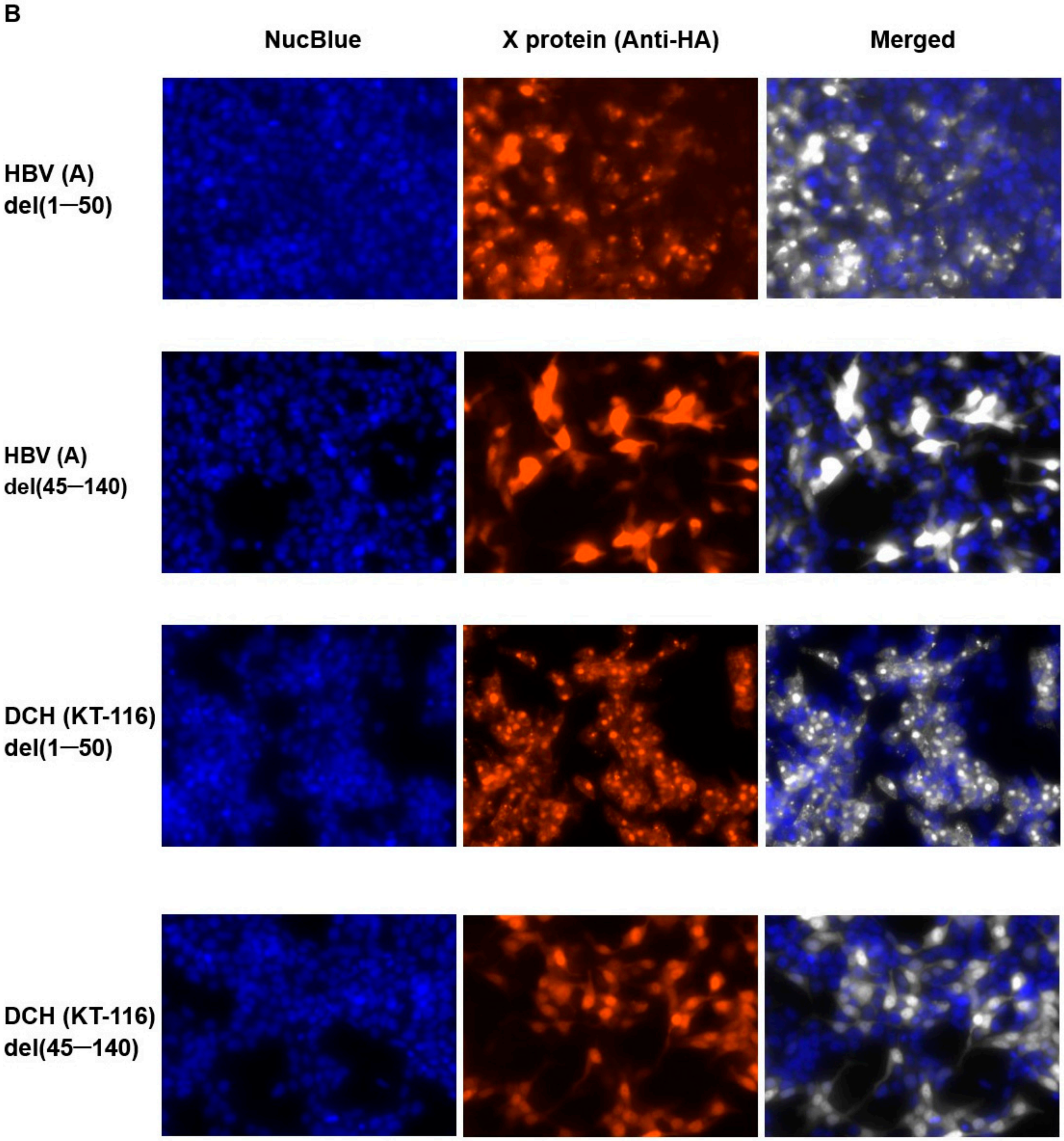

Supplementary Figure S4. Subcellular localization of *Orthohepadnavirus* X proteins in Lenti-X 293T cells. (A) A representative image of 40× magnification of Lenti-X 293T cells transfected with plasmids encoding *Orthohepadnavirus* X proteins. **(B)** A representative image of 40× magnification of Lenti-X 293T cells transfected with plasmids encoding del(1–50) or del(45–140) mutant X proteins derived from HBV (A) or DCH (KT-116). Cells were probed with Alexa Flour 647 anti-HA.11 (orange), and nuclei are stained with NucBlue (blue).

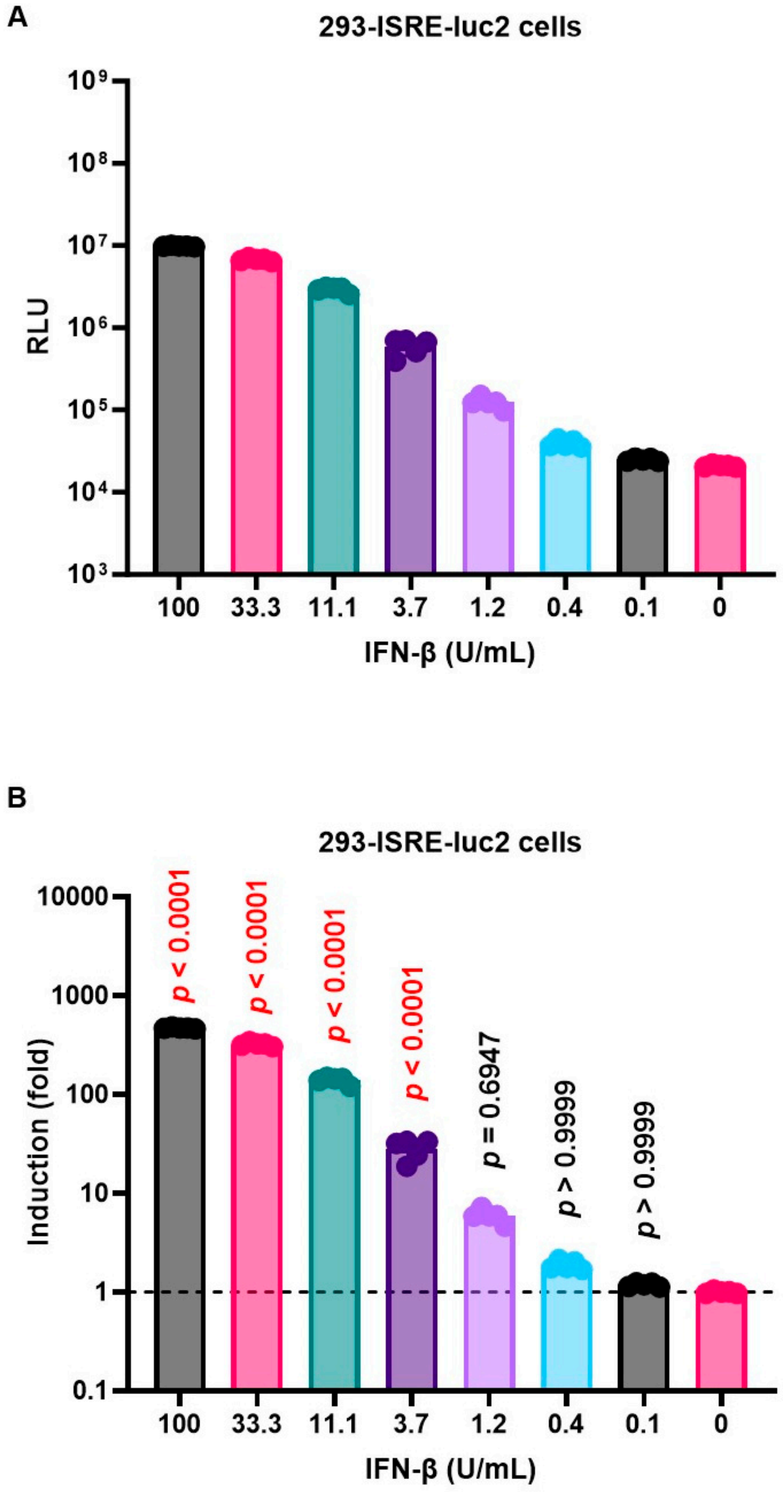

Supplementary Figure S5. Induction of luciferase reporter upon stimulation of 293-ISRE-luc2 cells with human IFN-β. (A) The RLU of 293-ISRE-luc2 cells stimulated with 0-100 U/mL of human IFN-β. **(B)** Induction of luciferase reporter upon stimulation of 293-ISRE-luc2 cells with human IFN-β. Differences between IFN-β-treated and control cells were examined by one-way ANOVA followed by Dunnett's multiple comparison test.

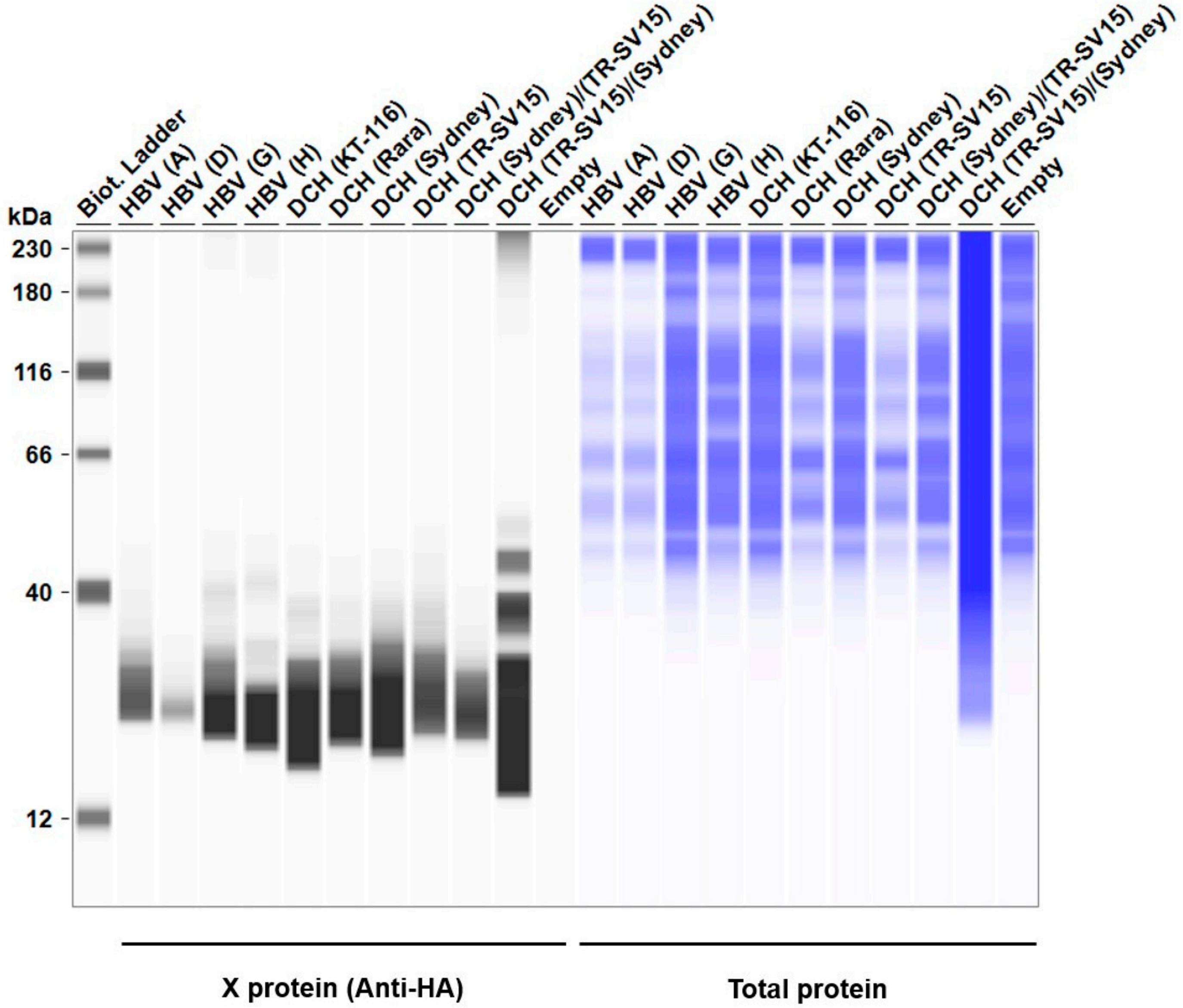

Supplementary Figure S6. Expression levels of *Orthohepadnavirus* X proteins in 293T-ISRE-luc2 cells.
